# The Global Molecular Prevalence of *Bartonella* spp. in Cats and Dogs: A Systematic Review and Meta-Analysis

**DOI:** 10.1155/2023/7867562

**Published:** 2023-11-08

**Authors:** Aya Attia Koraney Zarea, Maria Tempesta, Amienwanlen Eugene Odigie, Daniela Mrenoshki, Angela Fanelli, Vito Martella, Nicola Decaro, Grazia Greco

**Affiliations:** ^1^Department of Veterinary Medicine, University of Bari Aldo Moro, Bari, Italy; ^2^Department of Microbiology and Immunology, Veterinary Research Institute, National Research Centre, Dokki, Giza 12622, Egypt; ^3^Department of Veterinary Public Health and Preventive Medicine, University of Benin, Benin City 300238, Nigeria

## Abstract

*Bartonella* species are vector-borne infectious pathogens with a severe impact on animal and human health. This comprehensive systematic review aimed to perform a meta-analysis to evaluate the global impact of this pathogen on pet health. A literature search was performed on electronic databases (Web of Science, PubMed, and Scopus) to find relevant peer-reviewed published papers (*n* = 131). A random-effects model was employed to calculate pooled prevalence estimates, and *Q*-statistic and *I*^2^ index were used to assess the heterogeneity. Based on 20.133 cats and 9.824 dogs, the global prevalence estimates were 15.3% and 3.6%. The heterogeneity was significantly high in both species, with *I*^2^ = 95.8%, *p*-value <0.0001, and *I*^2^ = 87.7%, *p*-value <0.0001 in cats and dogs, respectively. The meta-analysis conducted using location coordinates showed a consistently high prevalence in regions located between latitudes −40 to −30 or latitudes 30–40 in both populations, in agreement with the pure spatial analysis results, which computed significantly high relative risk areas within these region coordinates. When analyzing cat data for other subgroup moderators, *Bartonella* spp. prevalence was higher in animals of young age (<1 year, *p*-value = 0.001), with a free roaming lifestyle (*p*-value <0.0001) and/or having ectoparasite infestation (*p*-value = 0.004). Globally, among the *Bartonella* species detected in cats, *Bartonella henselae* was the most frequent (13.05%), followed by *Bartonella clarridgeiae* (1.7%) and *Bartonella koehlerae* (0.11%). When considering *Bartonella henselae* genotype distribution, high heterogeneity (*p* < 0.0001) was observed based on geographical subgroups. Dogs displayed infection by *Bartonella vinsonii* subsp. *berkhoffii* (1.1%), *B. henselae* (1%), *Candidatus* B. merieuxii (0.9%) and *B. rochalimae* (0.38%). The present study provides a global picture of the epidemiological distribution of *Bartonella* spp. in cat and dog populations that may be pivotal for implementing proper preventive and control measures.

## 1. Introduction

*Bartonella* are fastidious small Gram-negative, facultative intracellular bacteria included in the alpha-2 subgroup of proteobacteria [1]. The genus *Bartonella* includes blood-borne vector-transmitted pathogens that infect a wide range of mammalian vertebrates, including wild and domestic carnivores [2–5]. Several *Bartonella* species and subspecies are recognized, with some of them confirmed as zoonotic and with companion animals reported as mammal reservoirs [6–9]. Cats act as primary reservoirs for the agents (i.e., *B. henselae*, *B. clarridgeiae*, and *B. koehlerae*) of human cat scratch disease (CSD) since they may have bacteremia persisting from weeks to years [2, 7, 10]. CSD is a worldwide zoonotic disease characterized by mild self-limiting signs to life-threatening syndromes, including endocarditis, meningitis, or encephalitis [2, 11–13]. Sporadically, cats can host other species, including *B. quintana*, *B. elizabethae*, *B. grahamii*, *B. bovis*, and *B. rochalimae* [4, 14–16]. Dogs host different *Bartonella* species, including *B. vinsonii* subsp. *berkhoffii*, *B. rochalimae*, and *C*. B. merieuxii [6, 11, 17–19]. In dogs, these bacterial species can induce asymptomatic infections or severe clinical manifestations, including endocarditis, splenomegaly, or vasculitis, as observed in humans [4, 7, 20, 21].

Currently, a rapid increase in the number of dogs and cats kept as family pets is being observed. Although companion dogs and cats provide their owners with substantial positive psychological and physiological benefits [22], they might act as sources of several pathogens, including zoonotic, thus posing a threat to human health [14, 23–25].

This study aimed to perform a systematic review of the existing literature reporting prevalence estimates of *Bartonella* spp. in cat and dog populations and to explore potential risk factors. Drawing a global picture of the risks posed by *Bartonella* spp. in pets is pivotal for implementing proper preventive and control measures under the One Health paradigm.

## 2. Materials and Methods

### 2.1. Protocol and Search Strategy

A systematic review was conducted with a meta-analysis of the studies on the molecular prevalence of *Bartonella* spp. and its determinants in cat and dog populations. Our protocol sticks to the guidelines of Preferred Reporting Items for Systematic Reviews and Meta-Analyses (PRISMA 2020) and the Statement protocol [26–28]. The research protocol is available in the International Prospective Register of Systematic Reviews (PROSPERO) under the Registration number CRD42022290813.

Three electronic databases, including Scopus (https://www.scopus.com/), Web of Science (https://apps.webofknowledge.com/), and PubMed (https://pubmed.ncbi.nlm.nih.gov) were investigated to retrieve relevant studies from their inception to November 2021. The search strategy was developed by combining the following descriptors (medical subject headings, MeSH) and their cross-referencing synonyms: (((“molecular” OR “survey” OR “occurrence” OR “prevalence”)) AND ((“*Bartonella ^*∗*^*” OR “bartonellosis” OR “*B. hensela”* OR “*B. clarridgeiae*” OR “*B. vinsonii subsp. Berkhoffii”* OR “*B. koehlerae*” OR “*Candidatus* Bartonella merieuxii” OR “*C. B*. merieuxii”)) AND ((“dog” OR “dogs” OR “domestic dogs” OR “*Canis lupus familiaris*” OR “canine” OR “Canis” OR “feline” OR “felines” OR “cat” OR “cats” OR “pets” OR “*Felis catus*” OR “domestic cat” OR “companion animals”))). After screening the matching studies, the relevant variables of interest were extracted for analysis [29].

### 2.2. Eligibility Criteria

**Figure 1:**
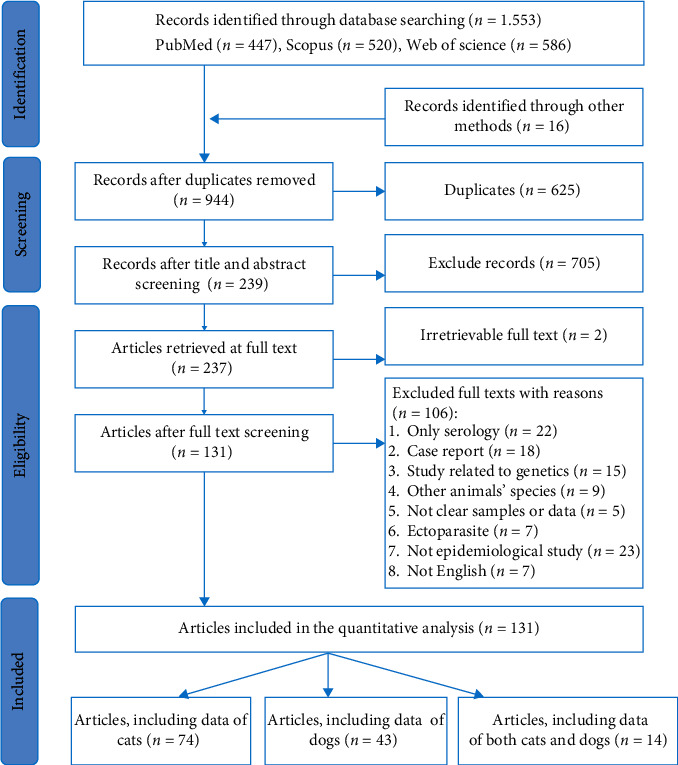
Flowchart for the selection of the studies included in the systematic review and meta-analysis of *Bartonella* infections in cat and dog populations.

The search was limited to the English language. Only studies based on molecular detection (polymerase chain reaction assays) of *Bartonella* spp. in cats and dogs consisting of observational research (cross-sectional) were included. Studies based on serological screening, case reports, clinical trials, workshop presentations, and opinion articles were excluded (Figure 1).

### 2.3. Selection of Studies and Reliability

Two reviewers (AZ and AEO) working independently considered the potential eligibility of each title and abstract based on the inclusion criteria (PICOTS Element). In case of disagreement, the third author (GG) judged and decided (the eligible articles were reviewed in full-text versions).

### 2.4. Data Extraction

Using a standardized data extraction form and working in duplicate, the following data were extracted from each study: (a) details of the publication (title, authors, and year); (b) full description of the enrolled animals, including species, timeframe of prevalence estimate (e.g., annual prevalence), animal population (cat or dog); (c) sample size; (d) prevalence estimates; (e) *Bartonella* species or variants (where available) detected; (f) possibly determinants for the *Bartonella* occurrence (e.g., age, gender, ectoparasite infestation, animal lifestyle, and geographic origin).

### 2.5. Risk of Bias (Quality) Assessment

Despite there are many specific tools available to assess the risk of bias in individual studies, these were not suitable for our research query. Thus, to minimize the risk of bias, a comment section was added to data extraction where the authors wrote any comments that could affect the interpretation of prevalence. Furthermore, the risk of bias was determined based on the sample selection process. Studies without sample selection criteria were considered at high risk of bias and removed from the analysis.

### 2.6. Summary Outcomes

The primary outcome was the pooled global molecular prevalence estimates of *Bartonella* spp. in cat and dog populations. A descriptive summary was constructed to present the results and the variations between studies according to the geographical subgroups. The further outcome was the variation of prevalence estimates of the *B. henselae* genotypes (based on 16S rRNA variability) in cat and dog populations on the basis of geographical subgroups. Location coordinates for each study and case were also extracted. Furthermore, moderators affecting the *Bartonella* spp. estimates were investigated.

### 2.7. Data Analyses and Synthesis of Results

For the eligible studies, meta-analysis was conducted using the Comprehensive Meta-Analysis (CME) software, version 3, following instructional guidelines [30]. Analyses were conducted for the two animal species separately. In case of more prevalence data reported from a single article (e.g., different host species and/or origin country), the information was considered as derived from separate studies.

The overall pooled prevalence of all sampled studies for each variable of interest was estimated with a random-effects meta-analysis of proportions using the Freeman–Turkey double arcine transformation with 95% exact confidence intervals [31–33]. Effect estimates and confidence intervals were presented in the forest plots. To obtain the 95% confidence interval, the random-effects model considers both within-study and between-study variability and assumes that the true effect sizes may vary across studies due to factors such as differences in study design, populations, or interventions. The 95% confidence interval provides a range within which can be 95% confident that the true effect size lies. The heterogeneity of the studies, expressed as the *I*-squared statistic, was assessed using a random-effects model [34], where the *I*-squared statistic represented the proportion of total variation in effects due to variation in true effects rather than sampling error.

#### 2.7.1. Spatial Statistics and Coordinate-Based Meta-Analyses

A retrospective purely spatial scan analysis to identify clusters with high likelihood probability was computed using SaTScan version 9.1.1 (SaTScan™ statistics software) using the Bernoulli probability model as described by Martin Kulldorff [35]. Clusters were outputted as a cylindrical window with a circular (or elliptical) geographic base, and the most likely cluster (based on likelihood-ratio test statistics) was identified as the principal cluster, which was reported when found to be statistically significant at *p* < 0.05. All statistically significant non-overlapping primary and secondary clusters were visualized using ArcGIS software (version 10.1). The relative risk (RR) statistic was used to assess the risk of feline and canine *Bartonella* infections in different spatial clusters. To obtain the RR, a comparison was made between the risk of *Bartonella* infection in the identified spatial clusters and a control group, which were negative for *Bartonella* spp. The RR was calculated by dividing the risk of feline and canine *Bartonella* infections in the exposed group (spatial clusters) by the risk of the control group.

A meta-analysis was conducted on all global studies retrieved using the location coordinates as a subgroup criterion. The analyses were performed on the samples based on two categories, namely countrywide and individual sample studies. The prevalence of *Bartonella* species was categorized into four geographic regions known as a first band (between latitudes −20 to 20), second band (between latitudes −30 to −20 or latitudes 20–30), third band (between latitudes −40 to −30 or latitudes 30–40) and fourth band (lesser than latitude −40 or greater than latitude 40), respectively.

The different *Bartonella* species occurrences and frequencies were geographically mapped using ArcGIS software (version 10.1).

### 2.8. Additional Analyses

#### 2.8.1. Geographical Distribution of the *Bartonella henselae* Genotypes

For *B. henselae*, prevalence estimates, mean effect sizes, and heterogeneity tests related to the geographical distribution of several genotypes were computed under the random effect models and expressed as odds ratios (OR).

#### 2.8.2. Moderator Analysis

The potential influences on *Bartonella* spp. prevalence estimates in cat populations were investigated using subgroup analyses ((gender, age class (≤ or >12 months old), ectoparasite infestation (yes or no), lifestyle (free-roaming or indoor), and geographic origin), and meta-regression. For dogs, due to the paucity of such studies and patchy information, the investigation for moderators was restricted to the geographic origin.

For each moderator, a random-effects model was built to combine studies within each subgroup; mean effect size and heterogeneity tests were computed and expressed as ORs.

All studies for which information about the subgroup was lacking were omitted from the analysis.

## 3. Results

### 3.1. Literature Investigation

As shown in Figure 1, a total of 1553 records were retrieved from the electronic databases and 16 from citation lists. After removing duplicates (*n* = 625), 944 papers were submitted for title and abstract screening. The reasons for exclusion are listed in the selection process flowchart (Figure 1). A total of 131 papers met our eligibility criteria. Out of the 131 included articles, 74 papers described *Bartonella* spp. occurrence in cats, 43 in dogs, and 14 in both the carnivore species. Furthermore, a unique study on dogs [36] was considered twice due to samples coming from different countries. Collectively, a total of 88 studies from cats and 58 from dogs were submitted to meta-analysis.

### 3.2. Risk of Bias

No relevant risk of bias was identified by the authors.

### 3.3. Geographical and Overtime Distribution of Studies

Since 1996, out of 88 studies retrieved reporting on *Bartonella* spp. prevalence estimates in cats, the highest number of studies were from Europe (*n* = 29, 33%), followed by the Americas (North, Central, and South) (*n* = 27, 30%), Asia (*n* = 22, 25%), Africa (*n* = 7, 8%), and Australia (*n* = 3, 3.4%) (Table S1). The annual frequency of studies retrieved in cats ranged from 1 to 9, with the highest number of studies performed during 2016 (*n* = 9), 2019, and 2021 (*n* = 8 per year). After one decade, surveys on *Bartonella* prevalence estimates in dog populations started, with Asia (*n* = 18, 31%) and America (*n* = 17, 29.4%) registering the highest number of studies, followed by Europe (*n* = 13, 22.4%) and Africa (*n* = 10, 17.2) (Table S2), with a peak of seven studies published in 2021.

### 3.4. Meta-Analysis of *Bartonella* spp. Prevalence Estimates Based on Individual Studies and Continent and Subcontinent Stratification

Eighty-eight and 58 prevalence estimates (from 74 and 43 articles) were included in the meta-analysis for cats and dogs, respectively (Tables S1 and S2). The pooled prevalence of *Bartonella* spp. based on 20,133 and 9,824 cats and dogs by using the random-effects model for individual studies was 15.03% and 3.6% (Figures S1 and S2), with significantly high heterogeneity, with values of *I*^2^ = 95.8%, *p*-value <0.0001 and *I*^2^ = 87.7%, *p*-value <0.0001 in cat and dog populations, respectively. Meta-regression analysis results of the *Bartonella* spp. prevalence estimates based on continent and subcontinent stratification for cat and dog populations are detailed in Tables S3 and S4, respectively. In feline populations, continent-wise meta-regression analysis revealed high heterogeneity (*I*^2^ = 81.29–99.16) with the prevalence estimates of *Bartonella* spp. being as high as 24.3% in studies conducted in the Americas (North, Central, and South), 16.6% in Asia, 15.8% in Australia, 14.5% in Europe, and 6.2% in Africa (Table S3).

In dog populations, continent-wise meta-regression analysis revealed high heterogeneity in America and Asia, while moderate and low heterogeneity was detected in Europe and Africa (Table S4). The *Bartonella* spp. prevalence was as high as 6% in the Americas, 4.2% in Asia, 2.8% in Africa, and 0.6% in Europe (Table S4).

#### 3.4.1. Meta-Analysis of Prevalence Estimates Based on Location Coordinates (Country and Sampling Site) and Spatial Distribution of Pure Spatial Clusters

The results of meta-regression analyses of the *Bartonella* spp. prevalence estimates based on countrywide location coordinates are shown in Figures S3 and S4 for cats and dogs, respectively. In addition, the results of the meta-analysis based on the location coordinates of the sampling area (individual study) for each animal host species (cat and dog) are detailed in Figures S5 and S6. The total feline *Bartonella* spp. prevalence for the first, second, third, and fourth bands were 15.1%, 6.6%, 24.8%, and 18.3%, respectively. Moreover, the total feline *Bartonella* spp. prevalence estimates based on the location coordinates of the collection sampling area (individual study) (Figure S5) for the first, second, third, and fourth bands were 5.1%, 6.9%, 8.3.0%, and 6.3%, respectively. A similar trend was also observed in the distribution of canine *Bartonella* infection across the four bands (Figures S4 and S6). Like what was observed for cats, the value registered in the third band was consistently higher in both categories (i.e., countrywide and sampling area) with an overall prevalence of 4.4% and 4.0%, respectively.

**Figure 2:**
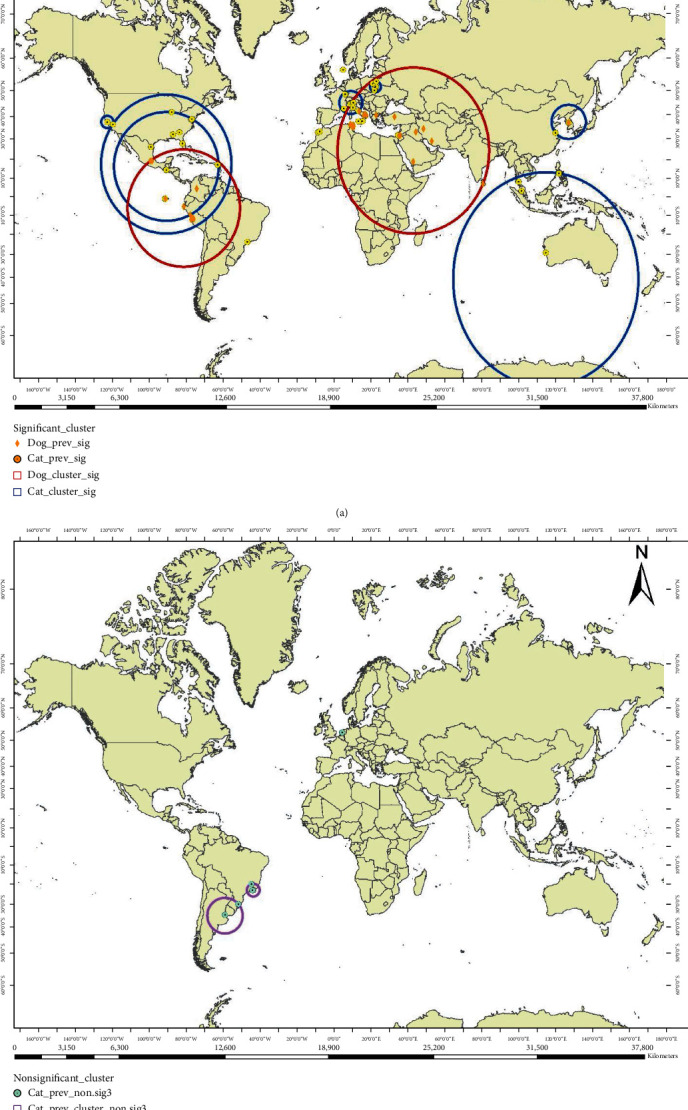
(a) Patterns of significant pure spatial scan statistic clusters (indicated by blue circles for cats and red circles for dogs) of *Bartonella* spp. infection. (b) Patterns of not significant clusters in cats.

Table S5 provides the characteristics of the statistically significant (*p* < 0.05) pure spatial scan statistic results. Fifteen and three significant clusters were identified for feline and canine *Bartonella* infections, respectively (Figure 2), Table S5. The RR for the identified spatial clusters for feline *Bartonella* infection ranged from 1.71 to 12.01 across approximate latitudes 57°N and 29°S, with the highest risk corresponding to regions located in the third band. In comparison, three significant clusters were calculated for canine *Bartonella* infection, with the RR ranging from 2.60 to 6.36 across regions lying between approximately latitudes 32°N to 38°S. Once again, the highest observed RR corresponded to regions of the third band. Comparatively, it revealed a tendency for increased overlap of feline and canine *Bartonella* infections in Europe and North and Central America, with nonsignificant clusters of feline *Bartonella* infection located in the regions of Brazil and Argentina in contrast with significant clusters spreading more north of the equator when compared with canine infections (Figure 2). However, this seems to be the reverse in Asia and Australia, as significant clusters occurred more south of the equator than observed in canine infections, with little or no overlap between both infections.

### 3.5. Total Prevalence of Different *Bartonella* Species Detected in Cats

**Figure 3:**
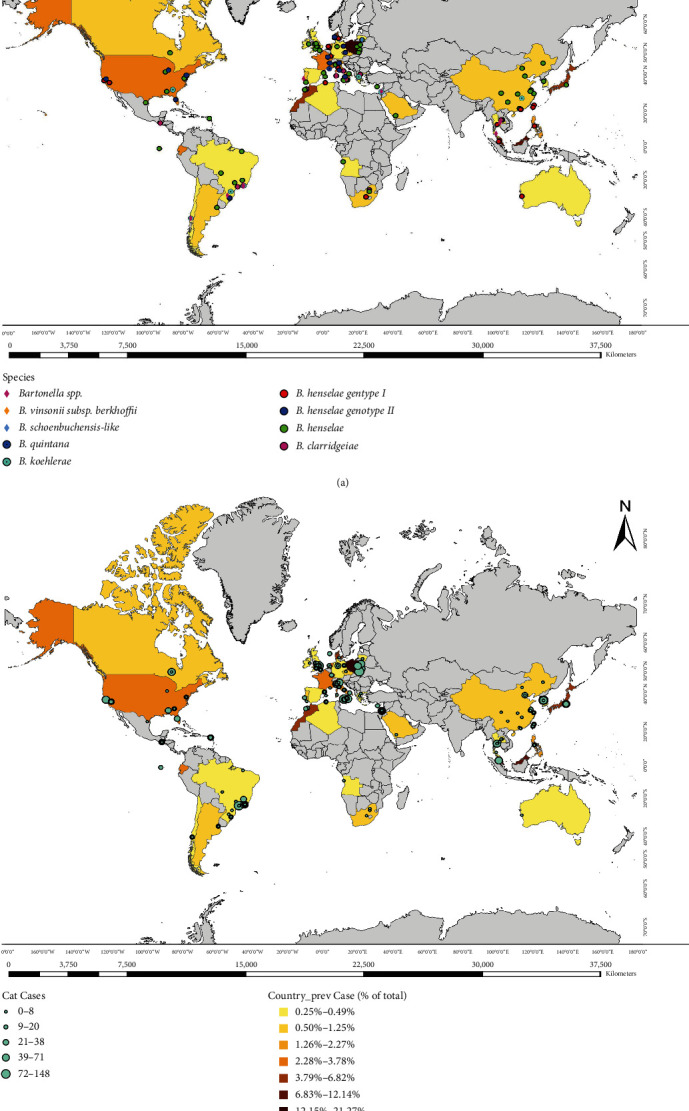
Global mapping of feline *Bartonella* spp. infections. (a) *Bartonella* species represented per sampling site coordinates. (b) Prevalence estimates of *Bartonella* spp. per country.

Out of a total of 20,133 cats, *B. henselae* was the predominant species, with a prevalence estimate of 11.08% (*n* = 2331), followed by *B. clarridgeiae* (1.7%, *n* = 345) and *B. koehlerae* (0.12% *n* = 24). Moreover, other species were sporadically detected from cats, such as *B. vinsonii* subsp. *berkhoffii* (*n* = 3) (Thailand and Chile) and *B. schoenbuchensis*-Like (*n* = 2) (Lithuania). Uncharacterized *Bartonella* species (1.5%, *n* = 293) and coinfections (0.21%, *n* = 42) were less common (Table S6).  Figure 3 shows the *Bartonella* species globally detected in cat populations according to the sampling site coordinates and the prevalence estimates per country.

### 3.6. Total Prevalence of Different *Bartonella* Species Detected in Dogs

**Figure 4:**
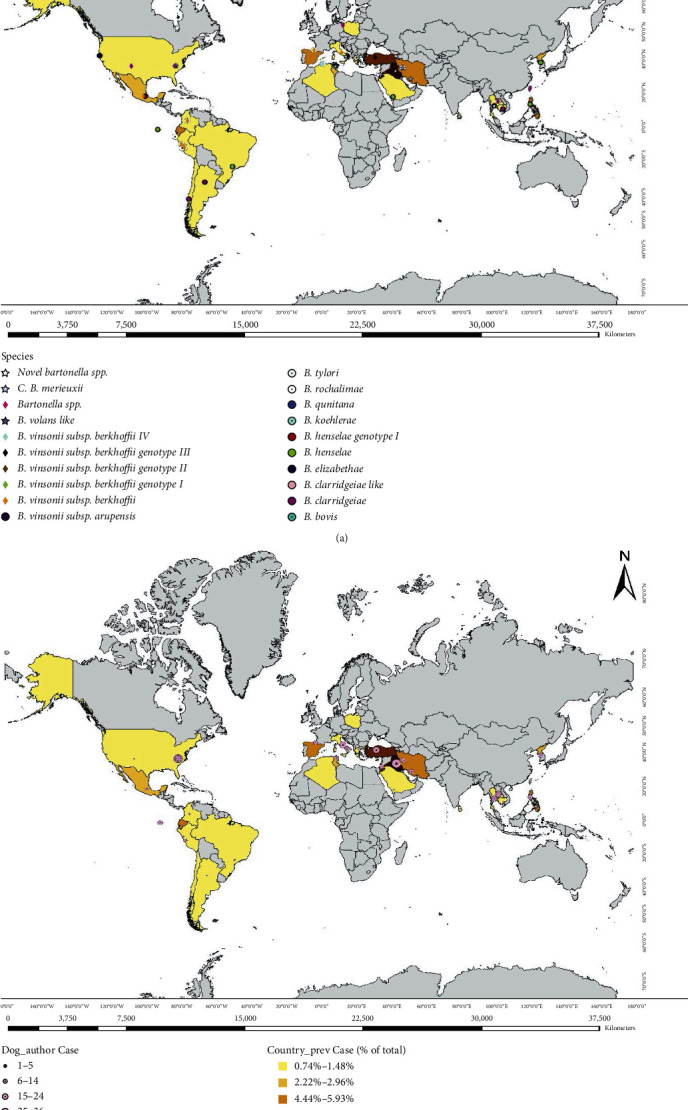
Global mapping of canine *Bartonella* spp. infections. (a) *Bartonella* species represented per sampling site coordinates. (b) Prevalence estimates of *Bartonella* spp. per country.

Out of 9,824 investigated dogs, *B. vinsonii* subsp. *berkhoffii* (*n* = 105, 1.07%), *B. henselae* (*n* = 73, 0.74%), and *C*.B. merieuxii (*n* = 96, 0.98%) were recorded with almost similar low frequencies, followed by *B. rochalimae* (*n* = 23, 0.23%), *B. clarridgeiae* (*n* = 23, 0.23%), *B. vinsonii* subsp. *arupensis* (*n* = 21, 0.21). Other *Bartonella* species included a novel species (<90% similarity to *B. vinsonii* subsp. *berkhoffii*) (*n* = 18, 0.18%), *B. elizabethae* (*n* = 15, 0.15%), *B. koehlerae* (*n* = 11, 0.11%), *B. taylorii* (*n* = 8, 0.08%), uncharacterized *Bartonella* spp. (*n* = 17, 0.16%), *B. volans*-like (*n* = 2), *B. bovis*, and *B. grahamii* (*n* = 1). Coinfections were also reported (*n* = 20, 0.20%) (Table S6). The global distribution of different *Bartonella* species according to the coordinates of the sampling sites is mapped in Figure 4.

### 3.7. Additional Analyses

#### 3.7.1. Analysis of the Spatial Distribution of *Bartonella henselae* Genotypes

On the basis of 25 (22 from cats and three from dogs) eligible studies (Table S7) reporting the occurrence of different *B. henselae* genotypes, high heterogeneity was observed according to geographical subgroups (*I*^2^ = 92.865, *p* < 0.0001), with *B. henselae* genotypes I (12%) more frequent than genotype II (1.7%) in Asia countries (Figure S7). On the opposite, in European countries *B. henselae* genotype II (9.6%) was more frequent than genotype I (5.4%). Both genotypes I and II were equally distributed in the Americas (7.4% and 6.8%, respectively) and Africa (6.2% and 4.1%, respectively) (Table S7, Figure S7).

#### 3.7.2. Moderator Analysis

A limited number of studies in cats detailing subgroup moderators (18 for lifestyle, 17 for age, 26 for sex, and 12 for osteoporosis) were available (Figure S8, panels (a)–(d)). The analyses for subgroup moderators revealed a significant difference in lifestyle (*I*^2^ = 83.36, *p*-value <0.0001), with stray cats being six times more at risk of exposure to *Bartonella* infection than cats living indoors (Table S8). In addition, there were significant differences in ectoparasite infestation (*I*^2^ = 62.4, *p*-value = 0.004) and age (<year; *I*^2^ = 44.89, *p*-value = 0.001). Cats infested with ectoparasites or younger than 1 year old had a two-fold higher risk for *Bartonella* spp. infection than non-infested or elderly cats, while no significant effect was observed for gender (Table S8, Figure S8). Due to the scarcity of reports on canine species, the analysis could not be conducted.

## 4. Discussion

*Bartonella* spp. infection represents a threat to the health of cats and dogs but also can impact public health. Therefore, mapping the risks posed by *Bartonella* spp. in cat and dog populations globally may be pivotal for implementing proper control measures. Studies summarizing published materials on cat and dog *Bartonella* infections are restricted to a few countries (i.e., California, Italy) [37, 38] or continent (Europe) [4]. The current systematic review and meta-analysis provide a comprehensive synthesis of evidence regarding the prevalence, diversity, and risk factors of *Bartonella* spp. infection in cats and dogs worldwide.

Molecular methods for *Bartonella* spp. have become the standard in diagnostics since culture-based methods are time-consuming and may not discriminate at or under the species level [39]. Accordingly, only relevant papers based on molecular diagnostics (PCR assays) were considered for the present study.

*Bartonella* spp. in cats was first identified in a 1996 study in Sydney [40] and subsequently in 1997 in studies from the Netherlands, New Zealand, and the USA [41–44]. Since then, the awareness of bartonellosis in cats has risen over time [4]. Furthermore, since dogs and cats share a vast repertoire of infectious pathogens [45], although with one-decade delay surveys for *Bartonella* spp. have been extended to dogs [4, 46, 47].

This meta-analysis study showed that the global pooled prevalence of *Bartonella* spp. was higher in cats (15.3%) compared to dogs (3.6%), with significantly higher heterogeneity being recorded in both species. In addition, in the cat populations, high heterogeneity was observed between different continents based on the meta-regression analysis with the highest pooled prevalence estimates of *Bartonella* spp. computed in the Americas (24.3%) (North, Central, and South) compared to Asia (16.6%), Australia (15.8%), Europe (14.5%), and Africa (6.2%). Nevertheless, the prevalence estimates for the various continental areas were based on previously published works, and hence, the interpretation of comparisons across regions should be made with caution due to the differences in the number of studies and the sampling size. Furthermore, being *Bartonella* spp. infection a no-mandatory reportable disease either in humans or animals, available epidemiological data are based on purely voluntary surveys. Out of the studies investigating the feline *Bartonella* spp. infection, the majority has been conducted in Europe, the Americas, and Asia compared to Africa and Australia. Southern European countries appeared to enact more intense molecular surveillance for *Bartonella* spp., with the majority of studies from Italy and Spain and with a subregional prevalence estimate of 8.4%, close to the estimates from Eastern Europe (8.2% in the Czech Republic) [48]. Noteworthy, Western Europe, which includes prevalence studies performed in the Netherlands [42], Denmark [49], Germany [50, 51], and France [15, 41, 52], accounted for the highest pooled *Bartonella* spp. prevalence (18.9%). A lower prevalence of 5.7% was documented in Northern Europe (Lithuania, Ireland, and the United Kingdom) [53–55]. As outgroups, studies from Poland and Portugal recorded the greatest (40.5%) and the lowest (2.9%) estimates of infection across the European continent, respectively [56–58]. Similarly, high interest in this topic was documented in the American continent, counting 27 studies (*p* = 24.3%), with the highest number from South America (66.6%, *p* = 26.1%), including Brazil (52%), Chile (7.4%) [59, 60] and Argentine (3.7%) [61]. The North American continent, based on studies from the USA and Canada [62–68], recorded lower pooled prevalence (22.3%) compared to Central areas, including the Caribbean Islands (Saint Kitts) and Guatemala (33.8%) [69, 70]. Studies from Asia recorded significantly high heterogeneity with the highest *Bartonella* spp. pooled prevalence estimates of 27.3% and 24.2% from Western Asia (Middle East) and Southeast Asia, respectively. Of interest, the Philippines had the highest *Bartonell*a spp. prevalence (61.3%) [43], followed by Turkey (40.1%) [71], Korea (33.6%) [72, 73] and Israel (28.6%) [3, 74]. Furthermore, other studies reported lower *Bartonella* prevalence rates varying between 9.1% and 19.6% in China [69, 75–77], Thailand [78–81], Saengsawang et al. [82], Taiwan [83, 84], Malaysia [85], Japan [86]; Sato et al. [87], Saudi Arabia [88].

Finally, Africa paid sparse surveillance on feline *Bartonella spp*. and restricted to a few countries from Northern-(Morocco and Algeria) [89–91] and Southern Africa (South Africa and Angola) [92–95], displaying pooled prevalence estimates of 19.2% and 3.8%, respectively. Similarly, there were only three studies from Oceania, in New Zealand and Australia, with prevalence estimates of 16.7% and 15.5%, respectively [40, 44, 96].

In dogs, compared to cats, there were a very low number of studies in the field, with the first case reports recorded in the early 2000s [97–99], followed by surveys performed in the USA and Brazil in 2007 [17, 100]. As observed in cats, continent-wise meta-analysis highlighted high heterogeneity in dog populations. The highest pooled *Bartonella* spp. prevalence estimates were recorded in the American continent (6%), followed by Asia (4.5%), Africa (2.8%), and Europe (0.6%). Asia recorded a high number of studies in dogs, with studies spread in Southeast, Western, and East Asia. The prevalence estimates through the countries varied between 0% to 37.1%, with the highest prevalence detected in free-ranging dogs in Iraq [18], Jordan [101], Iran [19, 102], Turkey [103], and China [76]. In the American continent, based on studies performed in southern and northern areas, the *Bartonella* spp. pooled prevalence estimates in dogs ranged between 4% and 8.4%. The USA recorded the highest number of studies in dogs, followed by Mexico, with pooled prevalence estimates of 8.3% and 12%, respectively. Prevalence estimates of *Bartonella* spp. ranged from 0.6% to 14.7% in South America, including studies from Brazil [17, 104], Chile [105, 106], Argentina [61, 107], Ecuador [108], Peru [109], and Colombia [109].

In Europe, Southern countries paid higher attention to this health topic than Central and Northern countries, recording low pooled *Bartonella* spp. prevalence estimates of 1.2%, 0.3%, and 0.1%, respectively. Country-wise investigations reported the highest prevalence of *Bartonella* spp. infection in rural or hunting dogs, compared to companion dogs, from Mediterranean countries, including Italy (5%) [36, 110–113] and Greece (4%) [36]. The prevalence was as low as 0.3% in Poland [114, 115] and was 0% in dogs from Portugal [116], Finland [117], and Spain [118, 119]. In the African continent, like what was observed for cats, studies were rare and mainly restricted to animals involved in governmental rabies control programs in Algeria [90, 120, 121] and Tunisia [122], with prevalence estimates of 4.4% to 14.8%. Surprisingly, *Bartonella* spp. was not detected in studies from Egypt [123], Morocco [91], Angola [95], Uganda [124], Maio Island, and Ghana [125].

In this meta-analysis study, it was shown that the prevalence estimates of Bartonella *spp*. infection globally varied in different geographical properties, including country. As *Bartonella* is a vector-borne transmitted pathogen, the factor “country” could be explicated with many factors, such as different local vector control measures and/or ecological contexts that can affect the spread rates of infection [126].

Furthermore, in the meta-analysis study based on both countrywide and individual studies (sampling site), location coordinates for the global distribution of feline and canine *Bartonella* spp. infection, it was shown a consistently higher prevalence (24.8%, 4.4%) in regions located at latitudes −40 to −30 or latitudes 30–40 (i.e., the third band) compared to regions lying in other latitudes. This agreed with the results of the space scan analyses that yielded statistically significant clusters for feline and canine *Bartonella* infection across approximate latitudes 57°N and 29°S and latitudes 32°N to 38°S, respectively, with the highest risk corresponding to regions located once again in the third band. Comparatively, there appears to be a correlation between the infection of *Bartonella* in cats and dogs, as the global distribution based on latitude coordinates followed the same pattern in both animal species. Once again, this may be due to a suitable ecological condition for the development and survival of the different arthropod vectors [127]. These findings provide a basis for further investigation on the possible reasons for this spatial behavior of *Bartonella* spp. infection, therefore, recommend investigating further components, including mean temperature, annual rainfall, sea level, and humidity, which can help elucidate possible predisposing factors, provide the evolution of infection over space and time, and thus guide improved control and prevention strategies.

The analysis for other subgroup moderators in cat populations revealed heterogeneity for ectoparasite infestation, free-roaming lifestyle, and age. The results of our meta-analysis significantly drive to the key role that ectoparasites play in the cycle of *Bartonella* infection since this study statistically provided that cats infested with ectoparasites had a two-fold higher risk of *Bartonella* infection. Indeed, fleas such as *Ctenocephalides felis* have a well-recognized active role in the transmission of *Bartonella* spp. that can multiply in flea stomachs [128–131]. Moreover, ticks have been proposed as vectors for the transmission of *Bartonella* species among cats, humans, dogs, and other mammalian hosts [132]. Furthermore, based on the present meta-analysis, cats with an outdoor lifestyle (pet or free-roaming) were six times more at risk to be bacteremic than indoor cats, with a further possible link to ectoparasite exposition. Indeed, the currently high permissiveness in some human communities, mainly in Western cultures, toward an outdoor lifestyle of dogs and cats, can perpetuate their exposition risk to ectoparasites and pathogens they transmit [133]. Finally, cat age significantly influenced the occurrence of *Bartonella* infections, with juvenile cats (less than 1 year) being two times more at risk than adults in terms of active infection by CSD agents, according to previous studies [126, 134–136]. Likely due to the scarcity or paucity of reports, the analysis for moderators in dog populations did not provide significant results.

A wide variety of *Bartonella* species, in most cases with known zoonotic potential, have been found to circulate among cats and dogs worldwide [6, 7, 9, 137–141]. Globally, *B. henselae* (13.05%) is the *Bartonella* species most frequently detected in cat populations, followed by *B. clarridgeiae* (1.7%) and *B. koehlerae* (0.11%) in the USA [66], Brazil [142, 143], Greece [144], and Australia [96]. Based on the variability of the 16S ribosomal DNA sequence and of *Pap*31gene, two main genotypes, designated as Huston I (genotype I) and Marseille II (genotype II), have been described in the *B. henselae* species [134, 145]. When considering the genotype distribution, high heterogeneity was observed according to geographical subgroups. The distribution of *B. henselae* types I (12%) and II (1.7%) in Asian countries differed from the distribution observed in European countries, where the type II (9.6%) was dominant compared to the type I (5.4%). Both genotypes were almost equally distributed in Africa (type I = 7.4% versus type II = 6.8%) and the Americas (type I = 6.2% versus type II = 4.1%). Of note, cats and humans can be infected with either genotype I or II and occasionally with a coinfection of both genotypes. In addition, types I and II seem to have different pathogenic roles in human bartonellosis, with type I appearing more pathogenic than type II [7, 146, 147], thus supposing a higher risk for human populations from Asia, where genotype I is more common.

*Bartonella* spp. is considered an emerging pathogen in dogs worldwide [6]. Dog is considered the primary host of *B. vinsonii* subsp. *berkhoffii*, *Candidatus* B. merieuxii, and possibly *B. rochalimae* [18]. Furthermore, dog can sporadically be infected with *B. henselae* and *B. koehlerae*, as reported in a few studies from Spain [148] and the USA [16, 149, 150].

*Bartonella vinsonii* subsp. *berkhoffii*, is the most frequently detected species in different (hunting, rural, or free-roaming) dog populations worldwide [17, 19, 36, 59, 103, 105, 109, 111, 112, 120, 148, 150–152] and encompasses four genotypes on the bases of the variability of the 16S-23S intergenic spacer region and the *Pap*31 gene [153]. Geographically, *B. vinsonii* subsp. *berkhoffii* genotype I was reported in Brazil and the USA [16, 17], the rare genotype II was detected in rural dogs from Italy [36], while the genotype III was more common globally, being detected in Iran, Turkey, Italy, Mexico, and USA [19, 100, 103, 112, 150, 152]. Finally, the rare genotype IV was reported only from two dogs in Algeria [120]. Collectively, *B. vinsonii* subsp. *berkhoffii* infection has been described in healthy dogs but often has been associated with endocarditis, arrhythmias, myocarditis, granulomatous lymphadenitis, and granulomatous rhinitis [4, 7, 20, 21].

*B. rochalimae*, an agent of splenomegaly in animals, was reported in dog populations with lower frequency (*p*=0.38%) [7, 19, 154, 155]. Furthermore, the HMD strain, later characterized as *C*. B. merieuxii, was reported in dogs from Italy and Greece [36]. Subsequently, *C*. B. merieuxii has been increasingly reported in domestic dogs and/or wild canids from Mediterranean and Middle East Asian countries, including Iraq [18], Tunisia [122], Iran [19, 102], Jordan [101], Italy [5] but rarely in Sri Lanka [109], and in a *Ct. felis* flea from an Indonesian dog [131]. Furthermore, wild canids, including coyotes, gray foxes, and wolves, have also been considered as possible reservoirs for both *B. vinsonii* subsp. *berkhoffii* and *B. rochalimae* [5, 7], thus representing a high threat at wild-domestic interfaces.

*Bartonella* species are highly adapted to one or more mammalian reservoir hosts and within which these bacteria have most probably coevolved to cause a long-lasting, relapsing, intraerythrocytic bacteremia [156]. Cats and dogs act as reservoir hosts for *B. hens*elae and *B. vinsonii* subsp. *berkhoffii*, respectively. Noteworthy, dogs and cats, yet sporadically, can be infected with the rodent *Bartonella* species as a result of their predation activities [133]. In detail, dogs from Thailand were infected with rodent *Bartonella* spp., including *B. vinsonii* subsp. *arupensis*, *B. elizabethae*, *B. grahamii*, and *B. taylorii* [157], while Pérez et al. [149] described *B. volans*-like in dogs from the USA. Likewise, *B. quintana*, the agent of trench fever that has humans as natural reservoirs [158], was described in cats from France [15] and dogs from Thailand [157]. Moreover, *Bartonella* species usually identified in ruminants, such as *B. schoenbuchensis* and *B. bovis*, were detected from cats in Lithuania [55] and from dogs in the USA [149]. Finally, un-characterizable *Bartonella* species were also reported in both species, thus highlighting the necessity for persisting surveys in the field.

## 5. Conclusion

Cats and dogs globally may host as a reservoir or accidentally a variety of *Bartonella* species, with cats significantly more likely to harbor these pathogens than dogs. Nevertheless, there appears to be a correlation between the infection of *Bartonella* in cats and dogs, as the global distribution based on coordinates followed the same pattern for both animal species. Indeed, this study identified spatial clusters for *Bartonella* infection across approximate latitudes −40 to −30 or latitudes 30–40 in both populations, revealing significantly higher risks within these region coordinates. Furthermore, the results of our meta-analysis significantly stress the central role of ectoparasites in the cycle of *Bartonella* infection since animals infested with ectoparasites had a two-fold higher risk of infection. Several *Bartonella* species from cats and dogs are zoonotic vector-borne pathogens; thus, mapping their global distribution in cat and dog populations and their risk factors may be pivotal for implementing proper control measures.

## Data Availability

The data supporting the findings of this study are included within the article and are available in the Supplementary Information Files.

## Supplementary Materials

Supplementary Materials Table S1: list of the studies (n.88) included in this study reporting the *Bartonella* spp. prevalence estimates of cat populations. Table S2: list of the studies (n.58) included in this study reporting the *Bartonella* spp. prevalence estimates of dogs' populations. Reference list of the studies submitted to meta-analyses as detailed in Tables S1 and S2. Table S3: meta-regression analyses of the prevalence estimates of *Bartonella* spp. in cat populations according to continent and subregion stratifications. Table S4: meta-regression analyses of the prevalence estimates of *Bartonella* spp. in dog populations according to continent and subregion stratifications. Table S5: summary of purely spatial scan statistics using the Bernoulli probability model (only significant clusters reported). Table S6: pooled prevalence estimates of *Bartonella* species according to animal host species and continent. Table S7: continental distribution of *B. henselae* genotypes detected from cats and dogs. Table 7.1: reference list of the studies reporting the genotype characterization of *B. henselae* detected in both cats and dogs and submitted to the analyses as reported in *Supplementary Table 7*. Table S8: analysis of *Bartonella* spp. infection in cats for subgroup moderators. Figure S1: forest plot of the random-effects meta-analysis of *Bartonella* prevalence estimates in cat populations for individual studies. Inverse variance index (*I*^2^ = 95.8%), variance between studies (*τ*^2^ = 0.913), *p*-value <0.0001, *Q*-value = 2064.9. Figure S2: forest plot of the random-effects meta-analysis of *Bartonella* prevalence estimates in dog populations for individual studies. Inverse variance index (*I*^2^ = 87.7%), variance between studies (*τ*^2^ = 1.186), *p*-value <0.0001, *Q*-value = 464.8. Figure S3: meta-analysis of *Bartonella* spp. prevalence estimates in cats based on the category of countrywide location coordinates. Figure S4: meta-analysis of *Bartonella* spp. prevalence estimates in dogs based on the category of countrywide location coordinates. Study/sampling site location coordinates. Figure S5: meta-analysis of *Bartonella* spp. from cats based on individual study/sampling site location coordinates. Figure S6: meta-analysis of *Bartonella* spp. from dogs based on the category of individual study/sampling site location coordinates. Figure S7: forest plot of the random-effect meta-analysis on the association of *B. henselae* genotypes I and II and continental distributions. Inverse variance index (*I*^2^ = 92.865), variance between studies (*τ*^2^ = 3.037), *p*-value <0.0001, *Q*-value = 168.186. Figure S8: panels (a)–(d): Forest plot of the random-effect meta-analysis on the association of *Bartonella* prevalence estimates and different moderators.
