# Supplementary material for: The Global Molecular Prevalence of Bartonella spp. in Cats and Dogs: A Systematic Review and Meta-Analysis
Source: Transbound Emerg Dis. 2023 Nov 8;2023:7867562. doi: 10.1155/2023/7867562 (PMC12017235; doi:10.1155/2023/7867562)

Supplementary information

1-LIST OF THE SUPPLEMENTARY TABLES

**Supplementary-Table 1:** List of the studies (n.88) included in this study reporting the *Bartonella* spp. prevalence estimates of cat populations.	**Pag. 3**
**Supplementary-Table 2:** List of the studies (n.58) included in this study reporting the *Bartonella* spp. prevalence estimates of dogs' populations.	**Pag. 7**
**Reference list of the studies submitted to meta-analyses as detailed in Tables S1 and S2**	**Pag. 10**
**Supplementary-Table 3:** Meta-regression analyses of the prevalence estimates of *Bartonella* spp. in cat populations according to continent and sub-region stratifications	**Pag. 20**
**Supplementary-Table**: Meta-regression analyses of the prevalence estimates of *Bartonella* spp. in dog populations according to continent and sub-region stratifications	**Pag. 21**
**Supplementary-Table 5:** Summary of purely spatial scan statistics using the Bernoulli probability model (only significant clusters reported).	**Pag. 22**
**Supplementary-Table 6:** Pooled prevalence estimates of *Bartonella* species according to animal host species and continent	**Pag. 23**
**Supplementary-Table 7.** Continental distribution of *B. henselae* genotypes detected from cats and dogs.	**Pag. 25**
**Supplementary-Table 7.1.** Reference list of the studies reporting the genotype characterization of *B. henselae* detected in both cats and dogs and submitted to the analyses as reported in the supplentary table 7.	**Pag. 26**
**Supplementary-Table 8:** Analysis of *Bartonella* spp. infection in cats for subgroup moderators.	**Pag. 29**

2-LIST OF THE SUPPLEMENTARY FIGURES

**Supplementary-Figure 1:** Forest plot of the random-effects meta-analysis of *Bartonella* prevalence estimates in cat populations for individual studies. Inverse variance index (I^2= 95.8%), variance between studies ($\tau 2$ =0.913), *p* value < 0.0001, Q-value = 2064.9.	**Pag. 30**
**Supplementary-Figure 2**: Forest plot of the random-effects meta-analysis of *Bartonella* prevalence estimates in dog populations for individual studies. Inverse variance index (I^2=87.7%), variance between studies ($\tau 2$ =1.186), *P*-value < 0.0001, Q-value = 464.8.	**Pag. 31**
**Supplementary-Figure 3.** Meta-analysis of *Bartonella* spp. prevalence estimates in cats based on category of countrywide location coordinates	**Pag. 32**
**Supplementary-Figure 4**: Meta-analysis of *Bartonella* spp. prevalence estimates in dogs based on category of countrywide location coordinates.	**Pag. 32**

study/sampling site location coordinates.	
**Supplementary-Figure 5:** Meta-analysis of *Bartonella* spp. from cats based on individual study/sampling site location coordinates. (reported as individual file due to the high size of the figure)	Add. file
**Supplementary-Figure 6:** Meta-analysis of *Bartonella* spp. from dogs based on category of individual study/sampling site location coordinates. (reported as individual file due to the high size of the figure)	Add. file
**Supplementary-Figure 7**: Forest plot of the random-effect meta-analysis on the association of *B. henselae* genotypes I and II and continental distributions. Inverse variance index (I^2=92.865), variance between studies (τ2 =3.037), *P*-value < 0.0001, Q-value = 168.186.	**Pag. 33**
**Supplementary Figure 8 Panels a-d:** Forest plot of the random-effect meta-analysis on the association of *Bartonella* prevalence estimates and different moderators.	**Pag. 34**

Supplementary Table 1: List of the studies (n.88) included in this study reporting *Bartonella* spp. prevalence estimates of cat populations.

**Author of study**	**CONTINENT/Country**	**Total**	**Cases**	**Prevalence**	**CI (95%)**	
					LB	UB
	**ASIA**					
Huang et al., 2019	China	164	25	15.2	9.74	20.74
Zhang, X. L et al., 2019	China	203	8	3.94	1.26	6.62
Zhang, Y et al., 2021	China	668	57	8.53	6.41	10.65
Yuan et al., 2011	China	359	46	12.8	9.35	16.27
Kim et al., 2009	Korea	146	59	40.41	32.45	48.37
Hwang et al., 2018	Korea	302	105	34.77	29.39	40.13
Satranarakun et al., 2017	Thailand	200	11	5.5	2.34	8.65
Inoue et al., 2009	Thailand	312	47	15.06	11.095	19.03
Saengsawang et al., 2021	Thailand	513	13	2.53	1.17	3.894
Srisanyong et al., 2016	Thailand	139	13	9.35	4.51	14.19
Gutiérrez et al., 2015	Israel	36	23	63.89	48.19	79.57
Gutiérrez et al., 2013	Israel	334	84	25.15	20.49	29.8
Chang et al., 2006	Taiwan	131	25	19.08	12.35	25.81
Tsai et al., 2011	Taiwan	103	21	20.39	12.61	28.17
Muz et al., 2021	Turkey	167	67	40.12	32.68	47.55
Hassan et al., 2017	Malaysia	284	48	16.9	12.54	21.26
Sato et al., 2017	Japan	1754	80	4.56	3.58	5.54
Alanazi et al., 2020	Saudi Arabia	44	4	9.09	0.59	17.58
Chomel et al.,1999	Philippines	31	19	61.3	44.14	78.44
Maruyama et al., 2000	Japan	690	50	7.25	5.31	9.18

Maruyama et al., 2001	Thailand	275	76	27.6	22.35	32.92
Assarasakorn et al., 2012	Thailand	153	26	16.99	11.04	22.94
	**AFRICA**					
Mesquita et al., 2021	Angola	66	2	3.03	0	7.16
Barradas et al., 2019	Angola	100	1	1	0	2.95
Bessas et al., 2016	Algeria	107	1	0.93	0	2.76
Azzag et al., 2012	Algeria	211	36	17.06	11.98	22.14
Chekli et al., 2020	Morocco	130	39	30	22.12	37.87
Lobetti, R., & Lappin ., 2012	South Africa	102	8	7.84	2.62	13.06
Pretorius et al., 1999	South Africa	31	1	3.22	0	9.44
	**AUSTRALIA**					
Joseph et al., 1997	New Zealand	48	8	16.67	6.12	27.21
Dybing et al., 2016	Australia	116	3	2.59	0	5.47
Branley et al., 1996	Australia	77	27	35.05	24.4	45.7
	**EUROPE**					
Razgūnaitė et al., 2021	Lithuania	163	8	4.91	1.59	8.22
Fabbi et al., 2004	Italy	769	140	18.21	15.47	20.9
Persichetti et al., 2016	Italy	42	16	38.09	23.41	52.8
Zobba et al.,2009	Italy	55	3	5.45	0	11.46
Persichetti et al., 2018	Italy	197	42	21.32	15.60	27.04
Ebani et al., 2012	Italy	234	24	10.26	6.37	14.14
Grippi et al., 2021	Italy	429	148	34.49	30	38.99
Otranto et al., 2017	Italy	330	13	3.94	1.84	6.038
Latrofa et al., 2020	Italy	958	24	2.501	1.51	3.49
Ebani et al., 2020	Italy	85	23	27.06	17.6	36.5
Angioni et al., 2020	Italy	52	7	13.46	4.18	22.74

Gracia et al., 2015	Spain	89	0	0	0	0
Ravicini et al.,2016	Spain	116	26	22.41	14.8	30
Tabar et al., 2008	Spain	100	1	1	0	2.95
Solano-Gallego et al., 2006	Spain	47	8	17.02	6.27	27.8
Millán et al., 2016	Spain	34	2	5.88	0	13.79
Arvand et al., 2001	Germany	193	20	10.36	6.06	14.66
Mietze et al., 2011	Germany	169	28	16.57	10.96	22.17
Heller et al., 1997	France	94	50	53.19	43.10	63.27
Rolain et al., 2004	France	99	8	8.08	2.712	13.45
Maia et al., 2014	Portugal	649	19	2.93	1.63	4.22
Melter et al., 2003	Czech Republic	61	5	8.19	1.31	15.08
Bergmans et al., 1997	Netherlands	113	25	22.1	14.47	29.77
Chaloner et al., 2011	UK	1782	103	5.78	4.69	6.86
Mazurek et al., 2020	Poland	672	272	40.5	36.76	44.19
Chomel et al., 2002	Denmark	93	21	22.6	14.08	31.08
Mylonakis et al.,2018	Greece	94	8	8.51	2.86	14.15
Juvet et al., 2010	Ireland	116	6	5.17	1.1	9.2
Attipa et al., 2017	Cyprus	174	19	10.9	6.28	15.55
	**AMERICA**					
Braga et al., 2012	Brazil	200	9	4.5	1.62	7.378
Furquim et al.,2021	Brazil	306	122	39.87	34.38	45.35
Pedrassani et al., 2019	Brazil	30	4	13.33	1.16	25.49
Miceli et al.,2013	Brazil	178	4	2.25	0.069	4.42
Silva et al., 2019	Brazil	89	22	24.7	15.75	33.68

Staggemeier et al., 2010	Brazil	47	8	17.02	6.27	27.76
Malheiros et al., 2016	Brazil	30	6	20	5.68	34.3
Raimundo et al., 2019	Brazil	208	83	39.9	33.24	46.56
André et al., 2016	Brazil	151	46	30.5	23.12	37.8
Braga et al., 2015	Brazil	182	3	1.6	0	3.49
Staggemeier et al., 2014	Brazil	47	12	25.53	13.06	37.9
Crissiuma et al., 2011	Brazil	40	17	42.5	27.18	57.82
De Bortoli et al., 2012	Brazil	46	2	4.35	0	10.24
André et al., 2014	Brazil	37	11	29.73	15	44.46
Müller et al., 2017	Chile	370	67	18.11	14.18	22.03
Sacristán et al .,2019	Chile	30	3	10	0	20.73
Cicuttin et al., 2014	Argentina	101	18	17.82	10.36	25.28
Levy et al., 2008	Ecuador	52	31	59.61	46.28	72.9
Huang et al., 2019	Caribbean (Stt. Kitts)	146	58	39.72	31.79	47.66
Kamrani et al., 2008	Canada	691	51	7.38	5.43	9.3
Lappin et al., 2006	Alabama	92	51	55.43	45.28	65.59
Hwang & Gottdenker et al., 2013	Georgia (St. Simons Island)	37	16	43.24	27.28	59.21
Guptill et al., 2004	California	271	65	23.98	18.9	29.06
Chomel et al.,1997	California	205	81	39.51	32.82	46.2
Fleischman et al., 2015	California	351	95	27.06	22.42	31.7
Bai et al., 2015	Guatemala	142	48	33.80	26.02	41.58
Shannon et al., 2017	Maryland (USA)	49	5	10.20	1.73	18.68

Supplementary Table 2: List of the studies (n.58) included in this study reporting *Bartonella* spp. prevalence estimates of dogs' populations.

**Author**	**Continent/Country**	**Total**	**Cases**	**Prevalence**	**CI (95%)**	
					**LB**	**UB**
	**ASIA**					
Kim et al., 2009 (Blood)	Korea	54	13	24.1	12.67	35.48
Suh et al., 2017	Korea	532	0	0	0	0
Samsami et al., 2020	Iran	98	12	12.4	5.75	18.73
Greco et al., 2019a	Iran	66	16	24.2	13.9	34.58
Singer et al., 2020	Philippine	160	6	3.75	0.81	6.69
Far et al., 2021	Jordan	80	14	17.5	9.17	25.83
Zhang, X. L et al., 2019	China	272	0	0	0	0
Tsai et al., 2011	Taiwan	120	2	1.7	0	3.96
Alanazi et al., 2020	Saudi Arabia	144	1	0.7	0	2.05
Chomel et al., 2012	Iraq	97	36	37.1	27.49	46.73
Ereqat et al., 2016	Palestine	110	1	0.91	0	2.68
Huggins et al., 2021	Cambodia	467	2	0.4	0	1.02
Celebi et al., 2010	Turkey	250	23	9.2	5.62	12.78
Billeter et al., 2012	Thailand	164	3	1.8	0	3.88
Saengsawang et al.,2021	Thailand	295	0	0	0	0
Bai et al., 2010	Thailand	192	60	31.25	24.69	37.81
Inoue et al., 2009	Thailand	350	1	0.3	0	0.84
Huggins et al., 2019	Thailand	100	1	1	0	2.95
	**AFRICA**					
Chekli et al., 2020	Morocco	153	0	0	0	0
Bessas et al., 2016	Algeria	117	1	0.85	0	2.52

Azzag et al., 2015	Algeria	96	7	7.29	2.09	12.49
Kernif et al., 2010	Algeria	80	5	6.25	0.94	11.55
Mesquita et al., 2021	Angola	19	0	0	0	0
Clarke et al., 2014	Ghana	17	0	0	0	0
Proboste et al., 2015	Uganda	38	0	0	0	0
Abdullah et al., 2021	Egypt	206	0	0	0	0
Belkhiria et al., 2017	Tunisia	149	22	14.8	9.06	20.46
Lauzi et al., 2016	Maio Island	153	0	0	0	0
	**EUROPE**					
Otranto et al., 2017	Italy	263	0	0	0	0
Otranto et al., 2010	Italy	109	6	5.5	1.2	9.78
Zobba et al., 2009	Italy	190	0	0	0	0
Diniz et al., 2009	Italy	60	7	11.7	3.54	19.78
Ebani et al., 2015	Italy	117	24	20.5	13.19	27.82
Millán et al., 2016	Spain	37	0	0	0	0
Tabar et al., 2009	Spain	153	0	0	0	0
Maia et al., 2015	Portugal	1010	0	0	0	0
Henriques et al., 2021	Portugal	61	0	0	0	0
Rymaszewska et al., 2011	Poland	242	1	0.41	0	1.22
Welc-Faleciak et al., 2009	Poland	82	0	0	0	0
Diniz et al., 2009	Greece	50	2	4	0	9.43
Pérez Vera et al., 2014	Finland	350	0	0	0	0
	**AMERICA**					
Diniz et al., 2007	Brazil	198	2	1.01	0	2.40

Silva et al., 2019	Brazil	124	0	0	0	0
Müller et al., 2018	Chile	139	6	4.3	0.93	7.7
Cevidanes et al., 2018	Chile	153	0	0	0	0
Kidd et al., 2017	California	42	0	0	0	0
Balakrishnan et al., 2014	North Carolina	118	21	17.8	10.89	24.7
Varanat et al., 2011	USA	137	28	20.4	13.68	27.2
Henn et al., 2007	California	182	4	2.19	0.07	4.3
Pérez et al., 2011	USA (several countries)	663	61	9.2	7	11.4
Diniz et al., 2010	Arizona	233	0	0	0	0
Arenas et al., 2019	Mexico	19	3	15.8	0	32.2
Tobar et al., 2020	Mexico	31	3	9.7	0	20.1
Levy et al., 2008	Ecuador	95	13	13.7	6.8	20.6
Diniz et al., 2013	Peru	205	21	10.3	6.1	14.4
Cicuttin et al., 2014	Argentina	94	0	0	0	0
Mascarelli et al., 2016	Argentina	66	2	3.03	0	7.2
Diniz et al., 2013	Colombia	22	2	9.1	0	21.1

List references included in the meta-analysis **(Tables S1 and S2)**

1. Abdullah, H., Amanzougaghene, N., Dahmana, H., Louni, M., Raoult, D., & Mediannikov, O. (2021). Multiple vector-borne pathogens of domestic animals in Egypt. PLoS neglected tropical diseases, 15(9), e0009767. https://doi.org/10.1371/journal.pntd.0009767
2. Alanazi, A. D., Alouffi, A. S., Alyousif, M. S., Alshahrani, M. Y., Abdullah, H., Abdel-Shafy, S., Calvani, N., Ansari-Lari, M., Sazmand, A., & Otranto, D. (2020). Molecular Survey of Vector-Borne Pathogens of Dogs and Cats in Two Regions of Saudi Arabia. Pathogens (Basel, Switzerland), 10(1), 25. https://doi.org/10.3390/pathogens10010025
3. André, M. R., Baccarim Denardi, N. C., Marques de Sousa, K. C., Gonçalves, L. R., Henrique, P. C., Grosse Rossi Ontivero, C. R., Lima Gonzalez, I. H., Cabral Nery, C. V., Fernandes Chagas, C. R., Monticelli, C., Alexandre de Santis, A. C., & Machado, R. Z. (2014). Arthropod-borne pathogens circulating in free-roaming domestic cats in a zoo environment in Brazil. Ticks and tick-borne diseases, 5(5), 545–551. https://doi.org/10.1016/j.ttbdis.2014.03.011
4. André, M. R., Dumler, J. S., Herrera, H. M., Gonçalves, L. R., de Sousa, K. C., Scorpio, D. G., de Santis, A. C., Domingos, I. H., de Macedo, G. C., & Machado, R. Z. (2016). Assessment of a quantitative 5' nuclease real-time polymerase chain reaction using the nicotinamide adenine dinucleotide dehydrogenase gamma subunit (nuoG) for *Bartonella* species in domiciled and stray cats in Brazil. Journal of feline medicine and surgery, 18(10), 783–790. https://doi.org/10.1177/1098612X15593787
5. Angioni, S. A., Di Gialleonardo, L., Di Domenico, M., Giansante, D., Tittarelli, M., & Cammà, C. (2020). Survey of Bartonella species in cats from Abruzzo region, Italy. Veterinaria italiana, 56(1), 10.12834/VetIt.1884.10006.2. https://doi.org/10.12834/VetIt.1884.10006.2
6. Arenas, P., Gil-Alarcón, G., Sánchez-Montes, S., Soto-Trujillo, M. P., Fernández-Figueroa, E., & Rangel-Escareño, C. (2019). Molecular detection of *Bartonella*, *Ehrlichia* and *Mycoplasma* in feral dogs of El Pedregal de San Angel Ecological Reserve in Mexico City. Revista brasileira de parasitologia veterinaria = Brazilian journal of veterinary parasitology: Orgao Oficial do Colegio Brasileiro de Parasitologia Veterinaria, 28(4), 728–734. https://doi.org/10.1590/S1984-29612019085
7. Arvand, M., Klose, A. J., Schwartz-Porsche, D., Hahn, H., & Wendt, C. (2001). Genetic variability and prevalence of *Bartonella henselae* in cats in Berlin, Germany, and analysis of its genetic relatedness to a strain from Berlin that is pathogenic for humans. Journal of clinical microbiology, 39(2), 743–746. https://doi.org/10.1128/JCM.39.2.743-746.2001
8. Assarasakorn, S., Veir, J. K., Hawley, J. R., Brewer, M. M., Morris, A. K., Hill, A. E., & Lappin, M. R. (2012). Prevalence of *Bartonella* species, hemoplasmas, and *Rickettsia felis* DNA in blood and fleas of cats in Bangkok, Thailand. Research in Veterinary Science, 93(3), 1213-1216. https://doi.org/10.1016/j.rvsc.2012.03.015
9. Attipa, C., Papasouliotis, K., Solano-Gallego, L., Baneth, G., Nachum-Biala, Y., Sarvani, E., Knowles, T. G., Mengi, S., Morris, D., Helps, C., & Tasker, S. (2017). Prevalence study and risk factor analysis of selected bacterial, protozoal and viral, including vector-borne, pathogens in cats from Cyprus. Parasites & vectors, 10(1), 130.https://doi.org/10.1186/s13071-017-2063-2
10. Azzag, N., Haddad, N., Durand, B., Petit, E., Ammouche, A., Chomel, B., & Boulouis, H. J. (2012). Population structure of *Bartonella henselae* in Algerian urban stray cats. PloS one, 7(8), e43621. https://doi.org/10.1371/journal.pone.0043621
11. Azzag, N., Petit, E., Gandoin, C., Bouillin, C., Ghalmi, F., Haddad, N., & Boulouis, H. J. (2015). Prevalence of select vector-borne pathogens in stray and client-owned dogs from Algiers. Comparative immunology, microbiology and infectious diseases, 38, 1–7. https://doi.org/10.1016/j.cimid.2015.01.001

12. Bai, Y., Kosoy, M. Y., Boonmar, S., Sawatwong, P., Sangmaneedet, S., & Peruski, L. F. (2010). Enrichment culture and molecular identification of diverse *Bartonella* species in stray dogs. Veterinary microbiology, 146(3-4), 314–319. https://doi.org/10.1016/j.vetmic.2010.05.017
13. Bai, Y., Rizzo, M. F., Alvarez, D., Moran, D., Peruski, L. F., & Kosoy, M. (2015). Coexistence of *Bartonella henselae* and *B. clarridgeiae* in populations of cats and their fleas in Guatemala. Journal of vector ecology: journal of the Society for Vector Ecology, 40(2), 327–332. https://doi.org/10.1111/jvec.12171
14. Balakrishnan, N., Musulin, S., Varanat, M., Bradley, J. M., & Breitschwerdt, E. B. (2014). Serological and molecular prevalence of selected canine vector borne pathogens in blood donor candidates, clinically healthy volunteers, and stray dogs in North Carolina. Parasites & vectors, 7, 116. https://doi.org/10.1186/1756-3305-7-116
15. Barradas, P. F., de Sousa, R., Vilhena, H., Oliveira, A. C., Luz, M. F., Granada, S., Cardoso, L., Lopes, A. P., Gonçalves, H., Mesquita, J. R., Ferreira, P., Amorim, I., & Gärtner, F. (2019). Serological and molecular evidence of *Bartonella henselae* in cats from Luanda city, Angola. Acta tropica, 195, 142–144. https://doi.org/10.1016/j.actatropica.2019.04.012
16. Belkhiria, J., Chomel, B. B., Ben Hamida, T., Kasten, R. W., Stuckey, M. J., Fleischman, D. A., Christopher, M. M., Boulouis, H. J., & Farver, T. B. (2017). Prevalence and Potential Risk Factors for *Bartonella* Infection in Tunisian Stray Dogs. Vector borne and zoonotic diseases (Larchmont, N.Y.), 17(6), 388–397. https://doi.org/10.1089/vbz.2016.2039
17. Bergmans, A. M., de Jong, C. M., van Amerongen, G., Schot, C. S., & Schouls, L. M. (1997). Prevalence of *Bartonella* species in domestic cats in The Netherlands. Journal of clinical microbiology, 35(9), 2256–2261. https://doi.org/10.1128/jcm.35.9.2256-2261.1997
18. Bessas, A., Leulmi, H., Bitam, I., Zaidi, S., Ait-Oudhia, K., Raoult, D., & Parola, P. (2016). Molecular evidence of vector-borne pathogens in dogs and cats and their ectoparasites in Algiers, Algeria. Comparative immunology, microbiology and infectious diseases, 45, 23–28. https://doi.org/10.1016/j.cmid.2016.01.002
19. Billeter, S. A., Sangmaneedet, S., Kosakewich, R. C., & Kosoy, M. Y. (2012). *Bartonella* species in dogs and their ectoparasites from Khon Kaen Province, Thailand. The Southeast Asian journal of tropical medicine and public health, 43(5), 1186–1192.
20. Braga, M., Diniz, P. P., André, M. R., Bortoli, C. P., & Machado, R. Z. (2012). Molecular characterisation of *Bartonella* species in cats from São Luís, state of Maranhão, north-eastern Brazil. Memorias do Instituto Oswaldo Cruz, 107(6), 772–777. https://doi.org/10.1590/s0074-02762012000600011
21. Braga, Í. A., Dias, I. S., Chitarra, C. S., Amude, A. M., & Aguiar, D. M. (2015). Molecular detection of *Bartonella clarridgeiae* in domestic cats from Midwest Brazil. The Brazilian journal of infectious diseases: an official publication of the Brazilian Society of Infectious Diseases, 19(4), 451–452. https://doi.org/10.1016/j.bjid.2015.05.002
22. Branley, J., Wolfson, C., Waters, P., Gottlieb, T., & Bradbury, R. (1996). Prevalence of *Bartonella henselae* bacteremia, the causative agent of cat scratch disease, in an Australian cat population. Pathology, 28(3), 262–265. https://doi.org/10.1080/00313029600169124
23. Celebi, B., Carhan, A., Kilic, S., & Babur, C. (2010). Detection and genetic diversity of *Bartonella vinsonii* subsp. *berkhoffii* strains isolated from dogs in Ankara, Turkey. The Journal of veterinary medical science, 72(8), 969–973. https://doi.org/10.1292/jvms.09-0466
24. Cevidanes, A., Di Cataldo, S., Vera, F., Lillo, P., & Millán, J. (2018). Molecular Detection of Vector-Borne Pathogens in Rural Dogs and Associated *Ctenocephalides felis* Fleas (Siphonaptera: Pulicidae) in Easter Island (Chile). Journal of medical entomology, 55(6), 1659–1663. https://doi.org/10.1093/jme/tjy141
25. Chaloner, G. L., Harrison, T. G., Coyne, K. P., Aanensen, D. M., & Birtles, R. J. (2011). Multilocus sequence typing of *Bartonella henselae* in the United Kingdom indicates that only a few, uncommon sequence types are associated with zoonotic disease. Journal of clinical microbiology, 49(6), 2132–2137. https://doi.org/10.1128/JCM.00275-11

26. Chang, C. C., Lee, C. C., Maruyama, S., Lin, J. W., & Pan, M. J. (2006). Cat-scratch disease in veterinary-associated populations and in its cat reservoir in Taiwan. Veterinary research, 37(4), 565–577. https://doi.org/10.1051/vetres:2006019
27. Chekli, Z., Haddad, N., Mellouki, F., Rhallabi, N., & Boulouis, H. J. (2020). First molecular detection of Bartonella spp. in stray cats and dogs in Morocco. International Journal of Infectious Diseases, 101, 543. https://doi.org/10.1016/j.ijid.2020.09.1408
28. Chomel, B. B., Boulouis, H. J., Gurfield, A. N., Heller, R., Piémont, Y., & Pilet, C. (1997). Maladie des griffes du chat et infections associées [Cat scratch disease and associated infections]. Bulletin de l'Academie nationale de medecine, 181(3), 441–454.
29. Chomel, B. B., Carlos, E. T., Kasten, R. W., Yamamoto, K., Chang, C. C., Carlos, R. S., Abenes, M. V., & Pajares, C. M. (1999). *Bartonella henselae* and *Bartonella clarridgeiae* infection in domestic cats from The Philippines. The American journal of tropical medicine and hygiene, 60(4), 593–597. https://doi.org/10.4269/ajtmh.1999.60.593
30. Chomel, B. B., Boulouis, H. J., Petersen, H., Kasten, R. W., Yamamoto, K., Chang, C. C., Gandoin, C., Bouillin, C., & Hew, C. M. (2002). Prevalence of *Bartonella* infection in domestic cats in Denmark. Veterinary research, 33(2), 205–213. https://doi.org/10.1051/vetres:2002008
31. Chomel, B. B., McMillan-Cole, A. C., Kasten, R. W., Stuckey, M. J., Sato, S., Maruyama, S., Diniz, P. P., & Breitschwerdt, E. B. (2012). *Candidatus* Bartonella merieuxii, a potential new zoonotic *Bartonella* species in canids from Iraq. PLoS neglected tropical diseases, 6(9), e1843. https://doi.org/10.1371/journal.pntd.0001843
32. Cicuttin, G. L., Brambati, D. F., De Gennaro, M. F., Carmona, F., Isturiz, M. L., Pujol, L. E., Belerenian, G. C., & Gil, H. (2014). *Bartonella* spp. in cats from Buenos Aires, Argentina. Veterinary microbiology, 168(1), 225–228. https://doi.org/10.1016/j.vetmic.2013.10.016
33. Clarke, L. L., Ballweber, L. R., Allen, K., Little, S. E., & Lappin, M. R. (2014). Prevalence of select vector-borne disease agents in owned dogs of Ghana. Journal of the South African Veterinary Association, 85(1), 996. https://doi.org/10.4102/jsava.v85i1.996
34. Crissiuma, A., Favacho, A., Gershony, L., Mendes-de-Almeida, F., Gomes, R., Mares-Guia, A., Rozental, T., Barreira, J., Lemos, E., & Labarthe, N. (2011). Prevalence of *Bartonella* species DNA and antibodies in cats (Felis catus) submitted to a spay/neuter program in Rio de Janeiro, Brazil. Journal of feline medicine and surgery, 13(2), 149–151. https://doi.org/10.1016/j.jfms.2010.08.010
35. De Bortoli, C. P., André, M. R., Seki, M. C., Pinto, A. A., Machado, S., & Machado, R. Z. (2012). Detection of hemoplasma and *Bartonella* species and co-infection with retroviruses in cats subjected to a spaying/neutering program in Jaboticabal, SP, Brazil. Revista brasileira de parasitologia veterinaria Brazilian journal of veterinary parasitology : Orgao Oficial do Colegio Brasileiro de Parasitologia Veterinaria, 21(3), 219–223. https://doi.org/10.1590/s1984-29612012000300008
36. Diniz, P. P., Schwartz, D. S., de Morais, H. S., & Breitschwerdt, E. B. (2007). Surveillance for zoonotic vector-borne infections using sick dogs from south-eastern Brazil. Vector borne and zoonotic diseases (Larchmont, N.Y.), 7(4), 689–697. https://doi.org/10.1089/vbz.2007.0129
37. Diniz, P. P., Billeter, S. A., Otranto, D., De Caprariis, D., Petanides, T., Mylonakis, M. E., Koutinas, A. F., & Breitschwerdt, E. B. (2009). Molecular documentation of *Bartonella* infection in dogs in Greece and Italy. Journal of clinical microbiology, 47(5), 1565–1567. https://doi.org/10.1128/JCM.00082-09
38. Diniz, P. P., Beall, M. J., Omark, K., Chandrashekar, R., Daniluk, D. A., Cyr, K. E., Koterski, J. F., Robbins, R. G., Lalo, P. G., Hegarty, B. C., & Breitschwerdt, E. B. (2010). High prevalence of tick-borne pathogens in dogs from an Indian reservation in north-eastern Arizona. Vector borne and zoonotic diseases (Larchmont, N.Y.), 10(2), 117–123. https://doi.org/10.1089/vbz.2008.0184
39. Diniz, P. P., Morton, B. A., Tngrian, M., Kachani, M., Barrón, E. A., Gavidia, C. M., Gilman, R. H., Angulo, N. P., Brenner, E. C., Lerner, R., & Chomel, B. B. (2013). Infection of domestic dogs in peru by zoonotic *Bartonella* species: a cross-sectional prevalence study of 219 asymptomatic dogs. PLoS neglected tropical diseases, 7(9), e2393. https://doi.org/10.1371/journal.pntd.0002393

40. Dybing, N. A., Jacobson, C., Irwin, P., Algar, D., & Adams, P. J. (2016). *Bartonella* Species Identified in Rodent and Feline Hosts from Island and Mainland Western Australia. Vector borne and zoonotic diseases (Larchmont, N.Y.), 16(4), 238–244.
41. Ebani, V. V., Bertelloni, F., & Fratini, F. (2012). Occurrence of *Bartonella* henselae types I and II in Central Italian domestic cats. Research in veterinary science, 93(1), 63–66. https://doi.org/10.1016/j.rvsc.2011.07.013
42. Ebani, V. V., Nardoni, S., Fognani, G., Mugnaini, L., Bertelloni, F., Rocchigiani, G., ... & Mancianti, F. (2015). Molecular detection of vector-borne bacteria and protozoa in healthy hunting dogs from Central Italy. *Asian Pacific Journal of Tropical Biomedicine*, *5*(2), 108-112. https://doi: 10.1016/S2221-1691(15)30153-2
43. Ebani, V. V., Guardone, L., Marra, F., Altomonte, I., Nardoni, S., & Mancianti, F. (2020). Arthropod-Borne Pathogens in Stray Cats from Northern Italy: A Serological and Molecular Survey. Animals: an open access journal from MDPI, 10(12), 2334. https://doi.org/10.3390/ani10122334
44. Ereqat, S., Nasereddin, A., Vayssier-Taussat, M., Abdelkader, A., Al-Jawabreh, A., Zaid, T., Azmi, K., & Abdeen, Z. (2016). Molecular Evidence of *Bartonella* Species in Ixodid Ticks and Domestic Animals in Palestine. Frontiers in microbiology, 7, 1217. https://doi.org/10.3389/fmicb.2016.01217
45. Fabbi, M., De Giuli, L., Tranquillo, M., Bragoni, R., Casiraghi, M., & Genchi, C. (2004). Prevalence of *Bartonella henselae* in Italian stray cats: evaluation of serology to assess the risk of transmission of Bartonella to humans. Journal of clinical microbiology, 42(1), 264–268. https://doi.org/10.1128/JCM.42.1.264-268.2004
46. Far, D., Takács, N., Gyurkovszky, M., Solymosi, N., & Farkas, R. (2021). Ticks and Tick-Borne Infections of Dogs in Two Jordanian Shelters. Vector borne and zoonotic diseases (Larchmont, N.Y.), 21(8), 573–578. https://doi.org/10.1089/vbz.2021.0026
47. Fleischman, D. A., Chomel, B. B., Kasten, R. W., Stuckey, M. J., Scarlet, J., Liu, H., Boulouis, H. J., Haddad, N., & Pedersen, N. C. (2015). *Bartonella* Infection among Cats Adopted from a San Francisco Shelter, Revisited. Applied and environmental microbiology, 81(18), 6446–6450. https://doi.org/10.1128/AEM.01864-15
48. Furquim, M. E. C., do Amaral, R., Dias, C. M., Gonçalves, L. R., Perles, L., Lima, C. A. P., Barros-Battesti, D. M., Machado, R. Z., & André, M. R. (2021). Genetic diversity and Multilocus Sequence Typing Analysis of Bartonella henselae in domestic cats from Southeastern Brazil. *Acta tropica*, *222*, 106037. https://doi.org/10.1016/j.actatropica.2021.106037
49. Gracia, M. J., Marcén, J. M., Pinal, R., Calvete, C., & Rodes, D. (2015). Prevalence of Rickettsia and Bartonella species in Spanish cats and their fleas. Journal of vector ecology: journal of the Society for Vector Ecology, 40(2), 233–239. https://doi.org/10.1111/jvec.12159
50. Greco, G., Sazmand, A., Goudarztalejerdi, A., Zolhavarieh, S. M., Decaro, N., Lapsley, W. D., Otranto, D., & Chomel, B. B. (2019a). High Prevalence of *Bartonella* sp. in Dogs from Hamadan, Iran. The American journal of tropical medicine and hygiene, 101(4), 749–752. https://doi.org/10.4269/ajtmh.19-0345
51. Grippi, F., Galluzzo, P., Guercio, A., Blanda, V., Santangelo, F., Sciortino, S., Vicari, D., Arcuri, F., Di Bella, S., & Torina, A. (2021). Serological and Molecular Evidence of *Bartonella* henselae in Stray Cats from Southern Italy. Microorganisms, 9(5), 979. https://doi.org/10.3390/microorganisms9050979
52. Guptill, L., Wu, C. C., HogenEsch, H., Slater, L. N., Glickman, N., Dunham, A., Syme, H., & Glickman, L. (2004). Prevalence, risk factors, and genetic diversity of *Bartonella henselae* infections in pet cats in four regions of the United States. Journal of clinical microbiology, 42(2), 652–659. https://doi.org/10.1128/JCM.42.2.652-659.2004
53. Gutiérrez, R., Morick, D., Gross, I., Winkler, R., Abdeen, Z., & Harrus, S. (2013). Bartonellae in domestic and stray cats from Israel: comparison of bacterial cultures and high-resolution melt real-time PCR as diagnostic methods. Vector borne and zoonotic diseases (Larchmont, N.Y.), 13(12), 857–864. https://doi.org/10.1089/vbz.2013.1308

54. Gutiérrez, R., Nachum-Biala, Y., & Harrus, S. (2015). Relationship between the Presence of *Bartonella* Species and Bacterial Loads in Cats and Cat Fleas (Ctenocephalides felis) under Natural Conditions. Applied and environmental microbiology, 81(16), 5613–5621. https://doi.org/10.1128/AEM.01370-15
55. Hassan, U. L., Dhaliwal, G. K., Watanabe, M., Ong, B. L., Yakubu, Y., & Tay, S. T. (2017). Feline bartonellosis associated with some clinicopathological conditions in a veterinary hospital in Selangor, Malaysia. Tropical bio medicine, 34(1), 174–179.
56. Heller, R., Artois, M., Xemar, V., De Briel, D., Gehin, H., Jaulhac, B., Monteil, H., & Piemont, Y. (1997). Prevalence of *Bartonella henselae* and *Bartonella clarridgeiae* in stray cats. Journal of clinical microbiology, 35(6), 1327–1331. https://doi.org/10.1128/jcm.35.6.1327-1331.1997
57. Henn, J. B., Gabriel, M. W., Kasten, R. W., Brown, R. N., Theis, J. H., Foley, J. E., & Chomel, B. B. (2007). Gray foxes (Urocyon cinereoargenteus) as a potential reservoir of a *Bartonella clarridgeiae*-like bacterium and domestic dogs as part of a sentinel system for surveillance of zoonotic arthropod-borne pathogens in northern California. Journal of clinical microbiology, 45(8), 2411–2418. https://doi.org/10.1128/JCM.02539-06
58. Henriques, J., Felisberto, R., Almeida, B., Ramos, J., Constantino-Casas, F., Dobson, J., Matos, R., Santos, A., de Sousa, R., & Alves, M. (2021). Canine lymphoma and vector-borne diseases: Molecular and serological evaluation of a possible complicity. Veterinary and comparative oncology, 19(1), 183–190. https://doi.org/10.1111/vco.12658
59. Huang, K., Kelly, P. J., Zhang, J., Yang, Y., Liu, W., Kalalah, A., & Wang, C. (2019). Molecular Detection of *Bartonella* spp. in China and St. Kitts. The Canadian journal of infectious diseases & medical microbiology Journal canadien des maladies infectieuses et de la microbiologie medicale, 2019, 3209013. https://doi.org/10.1155/2019/3209013
60. Huggins, L. G., Koehler, A. V., Ng-Nguyen, D., Wilcox, S., Schunack, B., Inpankaew, T., & Traub, R. J. (2019). Assessment of a metabarcoding approach for the characterisation of vector-borne bacteria in canines from Bangkok, Thailand. Parasites & vectors, 12(1), 394. https://doi.org/10.1186/s13071-019-3651-0
61. Huggins, L. G., Colella, V., Koehler, A. V., Schunack, B., & Traub, R. J. (2021). A multipronged next-generation sequencing metabarcoding approach unearths hyper diverse and abundant dog pathogen communities in Cambodia. Transboundary and emerging diseases, 10.1111/tbed.14180. Advance online publication. https://doi.org/10.1111/tbed.14180
62. Hwang, J., & Gottdenker, N. L. (2013). Bartonella species in raccoons and feral cats, Georgia, USA. Emerging infectious diseases, 19(7), 1167–1168. https://doi.org/10.3201/eid1907.130010
63. Hwang, J., Gottdenker, N. L., Oh, D. H., Nam, H. W., Lee, H., & Chun, M. S. (2018). Disentangling the link between supplemental feeding, population density, and the prevalence of pathogens in urban stray cats. Peer J, 6, e4988. https://doi.org/10.7717/peerj.4988
64. Inoue, K., Maruyama, S., Kabeya, H., Kawanami, K., Yanai, K., Jitchum, S., & Jittaparapong, S. (2009). Prevalence of Bartonella infection in cats and dogs in a metropolitan area, Thailand. Epidemiology and infection, 137(11), 1568–1573. https://doi.org/10.1017/S095026880900257X
65. Joseph, A. K., Wood, C. W., Robson, J. M., Paul, S. L., & Morris, A. J. (1997). *Bartonella henselae* bacteraemia in domestic cats from Auckland. New Zealand veterinary journal, 45(5), 185–187. https://doi.org/10.1080/00480169.1997.36023
66. Juvet, F., Lappin, M. R., Brennan, S., & Mooney, C. T. (2010). Prevalence of selected infectious agents in cats in Ireland. Journal of feline medicine and surgery, 12(6), 476–482. https://doi.org/10.1016/j.jfms.2010.02.003
67. Kamrani, A., Parreira, V. R., Greenwood, J., & Prescott, J. F. (2008). The prevalence of *Bartonella*, hemoplasma, and *Rickettsia felis* infections in domestic cats and in cat fleas in Ontario. Canadian journal of veterinary research. Revue canadienne de recherche veterinaire, 72(5), 411–419.
68. Kernif, T., Aissi, M., Doumandji, S. E., Chomel, B. B., Raoult, D., & Bitam, I. (2010). Molecular evidence of *Bartonella* infection in domestic dogs from Algeria, North Africa, by polymerase chain

reaction (PCR). The American journal of tropical medicine and hygiene, 83(2), 298–300. https://doi.org/10.4269/ajtmh.2010.09-0052

69. Kidd, L., Qurollo, B., Lappin, M., Richter, K., Hart, J. R., Hill, S., Osmond, C., & Breitschwerdt, E. B. (2017). Prevalence of Vector-Borne Pathogens in Southern California Dogs with Clinical and Laboratory Abnormalities Consistent With Immune-Mediated Disease. Journal of veterinary internal medicine, 31(4), 1081–1090. https://doi.org/10.1111/jvim.14735
70. Kim, Y. S., Seo, K. W., Lee, J. H., Choi, E. W., Lee, H. W., Hwang, C. Y., Shin, N. S., Youn, H. J., & Youn, H. Y. (2009). Prevalence of *Bartonella henselae* and *Bartonella clarridgeiae* in cats and dogs in Korea. Journal of veterinary science, 10(1), 85–87. https://doi.org/10.4142/jvs.2009.10.1.85
71. Lappin, M. R., Griffin, B., Brunt, J., Riley, A., Burney, D., Hawley, J., Brewer, M. M., & Jensen, W. A. (2006). Prevalence of *Bartonella* species, haemoplasma species, *Ehrlichia* species, *Anaplasma phagocytophilum*, and *Neorickettsia risticii* DNA in the blood of cats and their fleas in the United States. Journal of feline medicine and surgery, 8(2), 85–90. https://doi.org/10.1016/j.jfms.2005.08.003
72. Latrofa, M. S., Iatta, R., Toniolo, F., Furlanello, T., Ravagnan, S., Capelli, G., Schunack, B., Chomel, B., Zatelli, A., Mendoza-Roldan, J., Dantas-Torres, F., & Otranto, D. (2020). A molecular survey of vector-borne pathogens and haemoplasmas in owned cats across Italy. Parasites & vectors, 13(1), 116. https://doi.org/10.1186/s13071-020-3990-x
73. Lauzi, S., Maia, J. P., Epis, S., Marcos, R., Pereira, C., Luzzago, C., Santos, M., Puente-Payo, P., Giordano, A., Pajoro, M., Sironi, G., & Faustino, A. (2016). Molecular detection of *Anaplasma platys*, *Ehrlichia canis*, *Hepatozoon canis* and *Rickettsia monacensis* in dogs from Maio Island of Cape Verde archipelago. Ticks and tick-borne diseases, 7(5), 964–969. https://doi.org/10.1016/j.ttbdis.2016.05.001
74. Levy, J. K., Crawford, P. C., Lappin, M. R., Dubovi, E. J., Levy, M. G., Alleman, R., Tucker, S. J., & Clifford, E. L. (2008). Infectious diseases of dogs and cats on Isabela Island, Galapagos. Journal of veterinary internal medicine, 22(1), 60–65. https://doi.org/10.1111/j.1939-1676.2007.0034.x
75. Lobetti, R., & Lappin, M. R. (2012). Prevalence of *Toxoplasma gondii*, *Bartonella* species and haemoplasma infection in cats in South Africa. Journal of feline medicine and surgery, 14(12), 857–862. https://doi.org/10.1177/1098612X12452495
76. Maia, C., Ramos, C., Coimbra, M., Bastos, F., Martins, A., Pinto, P., Nunes, M., Vieira, M. L., Cardoso, L., & Campino, L. (2014). Bacterial and protozoal agents of feline vector-borne diseases in domestic and stray cats from southern Portugal. Parasites & vectors, 7, 115. https://doi.org/10.1186/1756-3305-7-115
77. Maia, C., Almeida, B., Coimbra, M., Fernandes, M. C., Cristóvão, J. M., Ramos, C., Martins, Â., Martinho, F., Silva, P., Neves, N., Nunes, M., Vieira, M. L., Cardoso, L., & Campino, L. (2015). Bacterial and protozoal agents of canine vector-borne diseases in the blood of domestic and stray dogs from southern Portugal. Parasites & vectors, 8, 138. https://doi.org/10.1186/s13071-015-0759-8
78. Malheiros, J., Costa, M. M., do Amaral, R. B., de Sousa, K., André, M. R., Machado, R. Z., & Vieira, M. (2016). Identification of vector-borne pathogens in dogs and cats from Southern Brazil. Ticks and tick-borne diseases, 7(5), 893–900. https://doi.org/10.1016/j.ttbdis.2016.04.007
79. Maruyama, Soichi, Nakamura, Y., Kabeya, H., Tanaka, S., Sakai, T., & Katsube, Y. (2000). Prevalence of *Bartonella henselae*, *Bartonella clarridgeiae* and the 16S rRNA Gene Types of *Bartonella henselae* among Pet Cats in Japan. Journal of Veterinary Medical Science, 62(3), 273–279. https://doi.org/10.1292/jvms.62.273
80. Maruyama, S., Sakai, T., Morita, Y., Tanaka, S., Kabeya, H., Boonmar, S., Poapolathep, A., Chalarmchaikit, T., Chang, C. C., Kasten, R. W., Chomel, B. B., & Katsube, Y. (2001). Prevalence of *Bartonella* species and 16s rRNA gene types of *Bartonella henselae* from domestic cats in Thailand. American Journal of Tropical Medicine and Hygiene, 65(6), 783–787. https://doi.org/10.4269/ajtmh.2001.65.783

81. Mascarelli, P. E., Tartara, G. P., Pereyra, N. B., & Maggi, R. G. (2016). Detection of *Mycoplasma haemocanis*, *Mycoplasma haematoparvum*, *Mycoplasma suis* and other vector-borne pathogens in dogs from Córdoba and Santa Fé, Argentina. Parasites & vectors, 9(1), 642. https://doi.org/10.1186/s13071-016-1920-8
82. Mazurek, Ł., Carbonero, A., Skrzypczak, M., Winiarczyk, S., & Adaszek, Ł. (2020). Epizootic Situation of Feline *Bartonella* Infection in Eastern Poland. Journal of veterinary research, 64(1), 79–83. https://doi.org/10.2478/jvetres-2020-0019
83. Melter, O., Hercík, K., Weyant, R. S., Janecek, J., Nemec, A., Mecera, J., Gonzorová, L., & Branny, P. (2003). Detection and characterization of feline Bartonella henselae in the Czech Republic. Veterinary microbiology, 93(3), 261–273. https://doi.org/10.1016/s0378-1135(03)00032-4
84. Mesquita, J. R., Oliveira, A. C., Neves, F., Mendoza, J. R., Luz, M. F., Crespo, I., Dos Santos, T. F., Santos-Silva, S., Vilhena, H., & Barradas, P. F. (2021). Hemotropic Mycoplasma and *Bartonella* Species Diversity in Free-Roaming Canine and Feline from Luanda, Angola. Pathogens (Basel, Switzerland), 10(6), 735. https://doi.org/10.3390/pathogens10060735
85. Miceli, N. G., Gavioli, F. A., Gonçalves, L. R., André, M. R., Sousa, V. R., Sousa, K. C., & Machado, R. Z. (2013). Molecular detection of feline arthropod-borne pathogens in cats in Cuiabá, state of Mato Grosso, central-western region of Brazil. Revista brasileira de parasitologia veterinaria = Brazilian journal of veterinary parasitology : Orgao Oficial do Colegio Brasileiro de Parasitologia Veterinaria, 22(3), 385–390. https://doi.org/10.1590/S1984-29612013000300011
86. Mietze, A., Morick, D., Köhler, H., Harrus, S., Dehio, C., Nolte, I., & Goethe, R. (2011). Combined MLST and AFLP typing of Bartonella henselae isolated from cats reveals new sequence types and suggests clonal evolution. Veterinary microbiology, 148(2-4), 238–245. https://doi.org/10.1016/j.vetmic.2010.08.012
87. Millán, J., Proboste, T., Fernández de Mera, I. G., Chirife, A. D., de la Fuente, J., & Altet, L. (2016). Molecular detection of vector-borne pathogens in wild and domestic carnivores and their ticks at the human-wildlife interface. Ticks and tick-borne diseases, 7(2), 284–290. https://doi.org/10.1016/j.ttbdis.2015.11.003
88. Müller, A., Walker, R., Bittencourt, P., Machado, R. Z., Benevenute, J. L., DO Amaral, R. B., Gonçalves, L. R., & André, M. R. (2017). Prevalence, hematological findings and genetic diversity of *Bartonella* spp. in domestic cats from Valdivia, Southern Chile. Parasitology, 144(6), 773–782. https://doi.org/10.1017/S003118201600247X
89. Müller, A., Soto, F., Sepúlveda, M., Bittencourt, P., Benevenute, J. L., Ikeda, P., Machado, R. Z., & André, M. R. (2018). *Bartonella vinsonii* subsp. *berkhoffii* and *B. henselae* in dogs. Epidemiology and infection, 146(9), 1202–1204. https://doi.org/10.1017/S0950268818001127
90. Muz, M. N., Erat, S., & Mumcuoglu, K. Y. (2021). Protozoan and Microbial Pathogens of House Cats in the Province of Tekirdag in Western Turkey. Pathogens (Basel, Switzerland), 10(9), 1114. https://doi.org/10.3390/pathogens10091114
91. Mylonakis, M. E., Schreeg, M., Chatzis, M. K., Pearce, J., Marr, H. S., Saridomichelakis, M. N., & Birkenheuer, A. J. (2018). Molecular detection of vector-borne pathogens in Greek cats. Ticks and tick-borne diseases, 9(2), 171–175. https://doi.org/10.1016/j.ttbdis.2017.08.013
92. Otranto, D., de Caprariis, D., Lia, R. P., Tarallo, V., Lorusso, V., Testini, G., Dantas-Torres, F., Latrofa, S., Diniz, P. P., Mencke, N., Maggi, R. G., Breitschwerdt, E., Capelli, G., & Stanneck, D. (2010). Prevention of endemic canine vector-borne diseases using imidacloprid 10% and permethrin 50% in young dogs: a longitudinal field study. Veterinary parasitology, 172(3-4), 323–332. https://doi.org/10.1016/j.vetpar.2010.05.017
93. Otranto, D., Napoli, E., Latrofa, M. S., Annoscia, G., Tarallo, V. D., Greco, G., Lorusso, E., Gulotta, L., Falsone, L., Basano, F. S., Pennisi, M. G., Deuster, K., Capelli, G., Dantas-Torres, F., & Brianti, E. (2017). Feline and canine leishmaniosis and other vector-borne diseases in the Aeolian Islands: Pathogen and vector circulation in a confined environment. Veterinary parasitology, 236, 144–151. https://doi.org/10.1016/j.vetpar.2017.01.019

94. Pedrassani, D., Biolchi, J., Gonçalves, L. R., Mendes, N. S., Zanatto, D., Calchi, A. C., Machado, R. Z., & André, M. R. (2019). Molecular detection of vector-borne agents in cats in Southern Brazil. Revista brasileira de parasitologia veterinaria. Brazilian journal of veterinary parasitology : Orgao Oficial do Colegio Brasileiro de Parasitologia Veterinaria, 28(4), 632–643. https://doi.org/10.1590/S1984-29612019077
95. Pérez Vera, C., Kapiainen, S., Junnikkala, S., Aaltonen, K., Spillmann, T., & Vapalahti, O. (2014). Survey of selected tick-borne diseases in dogs in Finland. Parasites & vectors, 7, 285. https://doi.org/10.1186/1756-3305-7-285
96. Pérez, C., Maggi, R. G., Diniz, P. P., & Breitschwerdt, E. B. (2011). Molecular and serological diagnosis of *Bartonella* infection in 61 dogs from the United States. Journal of veterinary internal medicine, 25(4), 805–810. https://doi.org/10.1111/j.1939-1676.2011.0736.x
97. Persichetti, M. F., Solano-Gallego, L., Serrano, L., Altet, L., Reale, S., Masucci, M., & Pennisi, M. G. (2016). Detection of vector-borne pathogens in cats and their ectoparasites in southern Italy. Parasites & vectors, 9(1), 247. https://doi.org/10.1186/s13071-016-1534-1
98. Persichetti, M. F., Pennisi, M. G., Vullo, A., Masucci, M., Migliazzo, A., & Solano-Gallego, L. (2018). Clinical evaluation of outdoor cats exposed to ectoparasites and associated risk for vector-borne infections in southern Italy. Parasites & vectors, 11(1), 136. https://doi.org/10.1186/s13071-018-2725-8
99. Pretorius, A. M., Kelly, P. J., Birtles, R. J., & Raoult, D. (1999). Isolation of *Bartonella henselae* from a serologically negative cat in Bloemfontein, South Africa. Journal of the South African Veterinary Association, 70(4), 154–155. https://doi.org/10.4102/jsava.v70i4.785
100. Proboste, T., Kalema-Zikusoka, G., Altet, L., Solano-Gallego, L., Fernández de Mera, I. G., Chirife, A. D., Muro, J., Bach, E., Piazza, A., Cevidanes, A., Blanda, V., Mugisha, L., de la Fuente, J., Caracappa, S., & Millán, J. (2015). Infection and exposure to vector-borne pathogens in rural dogs and their ticks, Uganda. Parasites & vectors, 8, 306. https://doi.org/10.1186/s13071-015-0919-x
101. Raimundo, J. M., Guimarães, A., Amaro, G. M., da Silva, A. T., Botelho, C., Massard, C. L., de Lemos, E., Favacho, A., & Baldani, C. D. (2019). Molecular Survey of *Bartonella* Species in Shelter Cats in Rio De Janeiro: Clinical, Hematological, and Risk Factors. The American journal of tropical medicine and hygiene, 100(6), 1321–1327. https://doi.org/10.4269/ajtmh.18-0585
102. Ravicini, S., Pastor, J., Hawley, J., Brewer, M., Castro-López, J., Beall, M., & Lappin, M. R. (2016). Prevalence of selected infectious disease agents in stray cats in Catalonia, Spain. JFMS open reports, 2(1), 2055116916634109. https://doi.org/10.1177/2055116916634109
103. Razgūnaitė, M., Lipatova, I., Paulauskas, A., Karvelienė, B., Riškevičienė, V., & Radzijevskaja, J. (2021). *Bartonella* Infections in Cats and Cat Fleas in Lithuania. Pathogens (Basel, Switzerland), 10(9), 1209. https://doi.org/10.3390/pathogens10091209
104. Rolain, J. M., Locatelli, C., Chabanne, L., Davoust, B., & Raoult, D. (2004). Prevalence of *Bartonella clarridgeiae* and *Bartonella henselae* in domestic cats from France and detection of the organisms in erythrocytes by immunofluorescence. Clinical and diagnostic laboratory immunology, 11(2), 423–425. https://doi.org/10.1128/cdli.11.2.423-425.2003
105. Rymaszewska, A., & Adamska, M. (2011). Molecular evidence of vector-borne pathogens coinfecting dogs from Poland. Acta veterinaria Hungarica, 59(2), 215–223. https://doi.org/10.1556/AVet.2011.008
106. Sacristán, I., Sieg, M., Acuña, F., Aguilar, E., García, S., López, M. J., Cevidanes, A., Hidalgo-Hermoso, E., Cabello, J., Vahlenkamp, T. W., Millán, J., Poulin, E., & Napolitano, C. (2019). Molecular and serological survey of carnivore pathogens in free-roaming domestic cats of rural communities in southern Chile. The Journal of veterinary medical science, 81(12), 1740–1748. https://doi.org/10.1292/jvms.19-0208
107. Saengsawang, P., Kaewmongkol, G., & Inpankaew, T. (2021). Molecular Detection of Bartonella spp. and Hematological Evaluation in Domestic Cats and Dogs from Bangkok, Thailand. Pathogens (Basel, Switzerland), 10(5), 503. https://doi.org/10.3390/pathogens10050503

108. Sato, S., Kabeya, H., Negishi, A., Tsujimoto, H., Nishigaki, K., Endo, Y., & Maruyama, S. (2017). Molecular survey of *Bartonella henselae* and *Bartonella clarridgeiae* in pet cats across Japan by species-specific nested-PCR. Epidemiology and infection, 145(13), 2694–2700. https://doi.org/10.1017/S0950268817001601
109. Satranarakun, P., Maruyama, S., Kabeya, H., Sato, S., Jitapalapong, S., Jitchum, S., Jiyipong, T., Rodkhum, C., & Pusoonthornthum, R. (2017). Prevalence of *Bartonella* infection in well-cared cats in Bangkok metropolitan. The Thai Journal of Veterinary Medicine, 46(4), 555–560. Retrieved from https://he01.tci-thaijo.org/index.php/tjvm/article/view/73776
110. Samsami, S., Ghaemi, M., & Sharifiyazdi, H. (2020). Molecular detection and phylogenetic analysis of '*Candidatus* Bartonella merieuxii' in dogs and its effect on hematologic parameters. Comparative immunology, microbiology and infectious diseases, 72, 101504. https://doi.org/10.1016/j.cimid.2020.101504
111. Shannon, A. B., Rucinsky, R., Gaff, H. D., & Brinkerhoff, R. J. (2017). *Borrelia miyamotoi*, Other Vector-Borne Agents in Cat Blood and Ticks in Eastern Maryland. EcoHealth, 14(4), 816–820. https://doi.org/10.1007/s10393-017-1268-3
112. Silva, B., Souza, A. M., Campos, S., Macieira, D. B., Lemos, E., Favacho, A., & Almosny, N. (2019). *Bartonella henselae* and *Bartonella clarridgeiae* infection, hematological changes and associated factors in domestic cats and dogs from an Atlantic rain forest area, Brazil. Acta tropica, 193, 163–168. https://doi.org/10.1016/j.actatropica.2019.02.026
113. Singer, G. A., Loya, F. P., Lapsley, W. D., Tobar, B. Z., Carlos, S., Carlos, R. S., Carlos, E. T., Adao, D., Rivera, W. L., Jaffe, D. A., Mazet, J., & Chomel, B. B. (2020). Detection of *Bartonella* infection in pet dogs from Manila, the Philippines. Acta tropica, 205, 105277. https://doi.org/10.1016/j.actatropica.2019.105277
114. Solano-Gallego, L., Hegarty, B., Espada, Y., Llull, J., & Breitschwerdt, E. (2006). Serological and molecular evidence of exposure to arthropod-borne organisms in cats from northeastern Spain. Veterinary microbiology, 118(3-4), 274–277. https://doi.org/10.1016/j.vetmic.2006.07.010
115. Srisanyong, W., Takhampunya, R., Boonmars, T., Kerdsin, A., & Suksawat, F. (2017). Prevalence of *Bartonella henselae*, *Bartonella clarridgeiae*, and *Bartonella vinsonii* subsp. *berkhoffii* in pet cats from four provincial communities in Thailand. The Thai Journal of Veterinary Medicine, 46(4), 663–670. Retrieved from https://he01.tci-thaijo.org/index.php/tjvm/article/view/73805
116. Staggemeier, R., Venker, C. A., Klein, D. H., Petry, M., Spilki, F. R., & Cantarelli, V. V. (2010). Prevalence of *Bartonella henselae* and *Bartonella clarridgeiae* in cats in the south of Brazil: a molecular study. Memorias do Instituto Oswaldo Cruz, 105(7), 873–878. https://doi.org/10.1590/s0074-02762010000700006
117. Staggemeier, R., Pilger, D. A., Spilki, F. R., & Cantarelli, V. V. (2014). Multiplex SYBR® green-real time PCR (qPCR) assay for the detection and differentiation of *Bartonella henselae* and *Bartonella clarridgeiae* in cats. Revista do Instituto de Medicina Tropical de Sao Paulo, 56(2), 93–95. https://doi.org/10.1590/S0036-46652014000200001
118. Suh, G. H., Ahn, K. S., Ahn, J. H., Kim, H. J., Leutenegger, C., & Shin, S. (2017). Serological and molecular prevalence of canine vector-borne diseases (CVBDs) in Korea. Parasites & vectors, 10(1), 146. https://doi.org/10.1186/s13071-017-2076-x
119. Tabar, M. D., Altet, L., Francino, O., Sánchez, A., Ferrer, L., & Roura, X. (2008). Vector-borne infections in cats: molecular study in Barcelona area (Spain). Veterinary parasitology, 151(2-4), 332–336. https://doi.org/10.1016/j.vetpar.2007.10.019
120. Tabar, M. D., Francino, O., Altet, L., Sánchez, A., Ferrer, L., & Roura, X. (2009). PCR survey of vector-borne pathogens in dogs living in and around Barcelona, an area endemic for leishmaniasis. The Veterinary record, 164(4), 112–116. https://doi.org/10.1136/vr.164.4.112
121. Tobar, B. Z., Lapsley, W. D., Swain, W. L., Jaffe, D. A., Setien, A. A., Galvez-Romero, G., Obregon-Morales, C., Olave-Leyva, J. I., & Chomel, B. B. (2020). *Bartonella* in dogs and fleas

from Tulancingo, Hidalgo, Mexico. Medical and veterinary entomology, 34(3), 302–308. https://doi.org/10.1111/mve.12438
122. Tsai, Y. L., Lin, C. C., Chomel, B. B., Chuang, S. T., Tsai, K. H., Wu, W. J., Huang, C. G., Yu, J. C., Sung, M. H., Kass, P. H., & Chang, C. C. (2011). *Bartonella* infection in shelter cats and dogs and their ectoparasites. Vector borne and zoonotic diseases (Larchmont, N.Y.), 11(8), 1023–1030. https://doi.org/10.1089/vbz.2010.0085
123. Varanat, M., Travis, A., Lee, W., Maggi, R. G., Bissett, S. A., Linder, K. E., & Breitschwerdt, E. B. (2009). Recurrent osteomyelitis in a cat due to infection with *Bartonella vinsonii* subsp. *berkhoffii* genotype II. Journal of veterinary internal medicine, 23(6), 1273–1277. https://doi.org/10.1111/j.1939-1676.2009.0372.x
124. Welc-Faleciak, R., Rodo, A., Siński, E., & Bajer, A. (2009). *Babesia canis* and other tick-borne infections in dogs in Central Poland. Veterinary parasitology, 166(3-4), 191–198. https://doi.org/10.1016/j.vetpar.2009.09.038
125. Yuan, C., Zhu, C., Wu, Y., Pan, X., & Hua, X. (2011). Bacteriological and molecular identification of *Bartonella* species in cats from different regions of China. PLoS neglected tropical diseases, 5(9), e1301. https://doi.org/10.1371/journal.pntd.0001301
126. Zhang, X. L., Li, X. W., Li, W. F., Huang, S. J., & Shao, J. W. (2019). Molecular detection and characterization of *Bartonella* spp. in pet cats and dogs in Shenzhen, China. Acta tropica, 197, 105056. https://doi.org/10.1016/j.actatropica.2019.105056
127. Zhang, Y., Zhang, Z., Lou, Y., & Yu, Y. (2021). Prevalence of hemoplasmas and *Bartonella* species in client-owned cats in Beijing and Shanghai, China. The Journal of veterinary medical science, 83(5), 793–797. https://doi.org/10.1292/jvms.20-0681
128. Zobba, R., Chessa, G., Mastrandrea, S., Pinna Parpaglia, M. L., Patta, C., & Masala, G. (2009). Serological and molecular detection of *Bartonell*a spp. in humans, cats and dogs from northern Sardinia, Italy. Clinical microbiology and infection: the official publication of the European Society of Clinical Microbiology and Infectious Diseases, 15 Suppl 2, 134–135. https://doi.org/10.1111/j.1469-0691.2008.02203.x

Supplementary Table 3: Meta-regression analyses of the prevalence estimates of *Bartonella* spp. in cat populations according to sub-region and continent stratifications

Continent	Sub-region	Studies (n)	Es	lower	Upper	Heterogeneity statistic (Q value)	P value	I^2
**Asia**	East Asia	10	0.046	0.055	0.33	329.6	0.001	99.1
	Southeast Asia	8	0.242	0.114	0.441	46.7	0.013	59.7
	Western Asia (Middle East)	4	0.273	0.168	0.41	15.29	0.002	86.9
	**Total**	**22**	**0.166**	**0.091**	**0.284**	**167.58**	**<0.0001**	**98.81**
**Africa**	North Africa	3	0.192	0.069	0.43	20.72	0.015	95.17
	South Africa	4	0.038	0.010	0.131	4.1	<.0001	75.6
	**Total**	**7**	**0.062**	**0.028**	**0.132**	**6.355**	**0.012**	**84.27**
**Australia**	Australia and New Zealand	3	0.158	0.117	0.209	0.036	n.r	0
**Europe**	Southern Europe	18	0.084	0.049	0.14	55.47	<0.0001	92.8
	Central Europe	1	0.405	0.368	0.442	0.00	n.r.	0.00
	Northern Europe	3	0.057	0.048	0.068	0.27	n.r	0.00
	Western Europe	6	0.189	0.125	0.274	31.04	<0.0001	87.11
	Eastern Europe	1	0.082	0.035	0.182	0.00	n.r	0
	**Total**	**29**	**0.145**	**0.069**	**0.279**	**475.8**	**<0.0001**	**99.16**
**America**	North America	8	0.223	0.084	0.473	131.4	0.032	98.5
	Central America	1	0.338	0.265	0.42	0.00	n.r.	0
	South America	18	0.261	0.171	0.378	40	<0.0001	92.5
	**Total**	**27**	**0.243**	**0.208**	**0.281**	**10.687**	**0.005**	**81.29**
**Global**		**88**	**0.153**	**0.127**	**0.182**	**2064.9**	**<0.0001**	**95.8**

Supplementary Table 4: Meta-regression analyses of the prevalence estimates of *Bartonella* spp. in dog populations according to continent and sub-region stratifications

Continent	Sub-region	Studies (n)	Es	lower	Upper	Heterogeneity statistic (Q value)	P value	I^2
**Asia**	East Asia	4	0.016	0.007	0.039	3.1	<0.0001	35.5
	Southeast Asia	7	0.026	0.008	0.08	14.7	<0.0001	86.4
	Western Asia	7	0.105	0.048	0.214	56.5	<0.0001	91.1
	**Total**	**18**	**0.045**	**0.016**	**0.121**	**90.35**	**<0.0001**	**97.79**
**Africa**	North Africa	6	0.031	0.009	0.102	26.26	<0.0001	48.8
	West Africa	1	0.00008	0.002	0.322	0.00	n.r.	0.00
	East Africa	2	0.00006	0.001	0.044	0.47	n.r.	0.00
	South Africa	1	0.00005	0.002	0.298	00	1	0.00
	**Total**	**10**	**0.028**	**0.01**	**0.077**	**4.248**	**0.236**	**29.38**
**Europe**	Southern Europe	10	0.012	0.002	0.063	15.27	<0.0001	80.36
	Central Europe	2	0.00003	0.00	0.022	0.00	n.r.	0.00
	Northern Europe	1	0.00001	0.000	0.022	0.00	n.r.	0.00
	**Total**	**13**	**0.006**	**0.001**	**0.034**	**6.51**	**0.04**	**69.28**
**America**	South America	9	0.04	0.016	0.098	37.83	<0.0001	86.77
	North America	8	0.084	0.71	0.1	0.849	n.r.	0
	**Total**	**17**	**0.06**	**0.030**	**0.117**	**17.292**	**<0.0001**	**94.22**
**Global**		**58**	**0.036**	**0.026**	**0.051**	**464.8**	**<0.0001**	**87.7**

Supplementary-Table 5: Summary of purely spatial scan statistics using the Bernoulli probability model (only significant clusters reported).

**Species**	**Radius (km)**	**latitude**	**longitude**	**case**	**population**	**RR**	**Case in area (%)**	**LLR**	***P*-value**
Cat	204.43	51.246 N	22.568 E	272	672	6.17	40.5	304.652	<0.00000001
	0.0	38.183 N	15.550 E	71	85	12.01	83.5	151.475	<0.00000001
	0.0	38.116 N	13.361 E	148	429	5.06	34.5	138.166	<0.00000001
	377.50	14.628 N	90.523 W	364	2092	2.65	17.4	132.060	<0.00000001
	813.78	37.460 N	126.95 E	173	646	3.94	26.8	119.912	<0.00000001
	3033.79	14.628 N	90.523 W	300	1645	2.74	18.2	117.498	<0.00000001
	0.0	23.534 S	46.625 W	106	380	4.03	27.9	76.647	<0.00000001
	483.05	45.465 N	9.189 E	239	1593	2.21	15.0	61.283	<0.00000001
	288.50	37.774 N	122.431 W	160	1101	2.10	14.5	37.273	<0.00000001
	16.27	32.109 N	34.855 E	109	776	2.01	14.0	22.985	<0.00000001
	0.0	12.880 N	121.774 E	23	62	5.25	37.1	22.854	<0.00000001
	0.0	3.509 N	101.525 E	48	284	2.40	16.9	15.413	0.000011
	4693.28	28.782 S	114.607 E	83	690	1.71	12.0	10.826	0.00091
	0.0	56.699 N	5.087 E	20	93	3.04	21.5	9.874	0.0023
	92.08	33.233 N	8.500 W	39	260	2.12	15.0	9.572	0.0031
Dog	979.28	32.423 N	54.127 E	64	261	6.36	24.5	66.9225	<0.00000001
	5730.25	37.258 N	99.647 W	156	1747	2.60	8.9	43.0201	<0.00000001
	0.0	33.790 N	8.8830 E	22	149	3.48	14.8	12.3488	0.000039

Supplementary-Table 6: Pooled prevalence estimates of *Bartonella* species according to animal host species and continent.

Bartonella spp.	Africa		Asia		America		Europe		Australia	Total	
	Cats<br>(747)	Dogs<br>(1028)	Cats<br>(7008)	Dogs<br>(3551)	Cats<br>(4128)	Dogs<br>(2521)	Cats<br>(8009)	Dogs<br>(2724)	Cats (241)	Cats<br>(20133)	Dogs<br>(9824)
B. henselae	46 (6.2)*<br>4.43-<br>7.88%	4 (0.4)<br>0.01<br>0.77%	737 (10.5)<br>9.8-11.2%	16 (0.45)<br>0.23- 0.67%	557 (13.5)<br>12.4-<br>14.54%	52 (2.1)<br>1.5- 2.62%	950 (11.9)<br>11.1-<br>12.57%	2 (0.07)<br>0.00-0.17%	37 (15.3)<br>10.8- 19.9%	2331 (11.08)<br>11.14-12.02%	73 (0.74)<br>0.57- 0.91%
B. clarridgeiae	3 (0.4)<br>0.00-<br>0.85%	1 (0.18)<br>0.00-<br>0.29%	136 (1.9)<br>1.6- 2.3%	9 (0.25)<br>0.09- 0.42%	127 (3.1)<br>2.55- 3.6%	12 (0.5)<br>0.21- 0.75%	79 (0.99)<br>0.77-<br>1.20%	1 (0.03)<br>0.00- 0.11%		345 (1.7)<br>1.53- 1.89%	23 (0.23)<br>0.14-0.33%
B. v subsp. berkhoffii		4(0.4)<br>0.01<br>0.77%	2 (0.03)<br>0.00-0.07%	28 (0.8)<br>0.58-1.08%	1 (0.02)<br>0.00-<br>0.07%	45 (1.8)<br>1.27- 2.3%		26 (0.95)<br>0.59-1.32%		3 (0.015)<br>0.00- 0.03%	105 (1.07)<br>0.87-1.27%
C B. merieuxii		22 (2.1)<br>1.3- 3.02%		65 (1.8)<br>1.4- 2.27%				7 (0.2)<br>0.07- 0.45%			96 (0.98)<br>0.78-1.17%
B. kholerae			11 (0.2)<br>0.06-,<br>0.25%		11 (0.3)<br>0.11-<br>0.42%	11 (0.4)<br>0.18- 0.69%	1 (0.01)<br>0.00-0.04%	1 (0.03)<br>0.00- 0.11%	1 (0.4)<br>0.00- 1.23%	24 (0.12)<br>0.07- 0.17%	11 (0.11)<br>0.05-0.18%
B. rochalimae		2 (0.28)<br>0.00-<br>0.46%		3 (0.08)<br>0.0- 0.18%		16 (0.6)<br>0.32- 0.94%		9 (0.3)<br>0.11- 0.55%			23 (0.23)<br>0.14- 0.33%
B. elizabethae		2 (0.2)<br>0.00-<br>0.46%		9 (0.2)<br>0.09- 0.42%		4 (0.15)<br>0.003- 0.31%					15 (0.15)<br>0.07- 0.23%
B. schoenbuchensis -<br>like							2 (0.02)<br>0.00-0.06%			2 (0.018)<br>0.00-0.02%	
B. v. subsp. *aurpensis*				21 (0.6)<br>0.34- 0.84%							21 (0.21)<br>0.12- 0.31%
Novel *Bartonella* spp				18 (0.5)<br>0.27- 0.74%							18 (0.18)<br>0.09-0.27%
B. taylorii				8 (0.2)<br>0.07- 0.38%						8 (0.08)<br>0.02- 0.14%	
B. grahamii				1 (0.03)<br>0.00-0.08%							
B. bovis						1 (0.04)<br>0.00-0.12%					

B. volans -like						2 (0.08)<br>0.00-0.19%					
Bartonella spp.	39 (5.2)<br>3.6- 6.8%			8 (0.2)<br>0.07- 0.38%	237 (5.7)<br>5.03-<br>6.45%	8 (0.3)<br>0.09- 0.54%	19 (0.24)<br>0.13-<br>0.34%	1 (0.03)<br>0.00- 0.11%	293 (1.5)<br>1.29- 1.62%	17 (0.16)<br>0.09-0.25%	
Co-infection			36 (0.5)<br>0.35-<br>0.68%	3 (0.08)<br>0.00- 0.18%	5 (0.1)<br>0.01-,<br>0.23%	15 (0.6)<br>0.29- 0.89%	1 (0.01)<br>0.00-0.04%		42 (0.21)<br>0.15-0.27%	20 (0.20)<br>0.11- 0.29%	

Legend:*= positive (%) CI 95%

Supplementary table 7. Continental and sub-regional distribution of *B. henselae* genotypes detected from cats and dogs

Continent	Sub-region	N. of studies	Total N. of animals	*B. henselae* positive cases n (%)	CI 95%	*B. henselae* genotypes n (%), CI 95%					
						Bh I	CI 95%	Bh II	CI 95%	Co-infection	CI 95%
EUROPE	Western *	5	592	106 (17.9)	14.82, 20.99	36 (6.1)	4.16, 8.0	70 (11.8)	9.22, 14.43	n.r.	-
	Eastern	1	61	5 (8.2)	1.31, 15.08	n.r.	-	5 (8.2)	1.31, 15.08	n.r.	-
	Southern	3	1088	178 (16.4)	14.16, 18.56	59 (5.4)	4.08, 6.77	93 (8.5)	6.89, 10.21	26 (2.3)	1.48, 3.30
Total		9	1741	289 (16.6)	14.85, 18.35	95 (5.4)	4.39, 6.52	168 (9.6)	8.26, 11.04	26 (1.5)	0.92, 2.06
ASIA	East	3	437	51 (11.7)	8.66, 14.68	25 (5.7)	3.54, 7.90	14 (3.2)	1.55, 4.85		
	Southeast	3	627	108 (17.2)	14.27, 20.18	103 (16.4)	13.53, 19.33	4 (0.64)	0.015, 1.261	1 (0.16)	0.00, 0.47
Total		6	1064	159 (14.9)	12.80, 17.09	128 (12)	10.08, 13.98	18 (1.7)	0.92%, 2.47	13 (1.2)	0.56, 1.88
AFRICA	North	1	211	30 (14.2)	9.51, 18.93	14 (6.6)	3.28, 9.99	10 (4.7)	1.87, 7.61	6 (2.8)	0.60, 5.09
	South	1	31	1 (3.2)	0.00, 9.45	1 (3.2)	0.00, 9.45	n.r.	-	n.r.	-
Total		2	242	31 (12.8)	8.60, 17.02	15 (6.2)	3.16, 9.24	10 (4.1)	1.62, 6.64	6 (2.5)	0.52, 4.44
AMERICA	North	4	771	158 (20.49)	17.64, 23.34	57 (7.39)	5.55, 9.24	86 (11.15)	8.93, 13.38	15 (1.95)	0.97, 2.92
	Central	1	142	32 (22.5)	15.66, 29.41	32 (22.5)	15.66, 29.41	n.r.	-	n.r.	-
	South	3	466	21(4.71)	2.74, 6.67	13(2.91)	1.35, 4.48	8 (1.79)	0.56, 3.03	n.r	-
Total		8	1379	211 (15.3)	13.40, 17.20	102 (7.4)	6.02, 8.78	94 (6.8)	5.49, 8.15	15 (1.09)	0.54, 1.64

Bh I: *B. henselae* genotype I, Bh II: *B. henselae* genotype II
n.r.: not reported

Categorization of the countries*: Europe: Western (France, Netherlands, Germany, Denmark), Eastern (Czech Republic), South (Italy)
Asia: East (Taiwan, China), Southeast (Thailand, Malaysia, Philippines)
Africa: North (Algeria), South (South Africa)
America: North (USA, Mexico), Central (Guatemala) and South (Chile, Argentina).

Supplementary Table 7.1. Reference list of the studies reporting the genotype characterization of *B. henselae* detected in both cats and dogs and submitted to the analyses as reported in the supplentary table 7.

Arvand, M., Klose, A. J., Schwartz-Porsche, D., Hahn, H., & Wendt, C. (2001). Genetic variability and prevalence of *Bartonella henselae* in cats in Berlin, Germany, and analysis of its genetic relatedness to a strain from Berlin that is pathogenic for humans. Journal of clinical microbiology, 39(2), 743–746. https://doi.org/10.1128/JCM.39.2.743-746.2001
Azzag, N., Haddad, N., Durand, B., Petit, E., Ammouche, A., Chomel, B., & Boulouis, H. J. (2012). Population structure of *Bartonella henselae* in Algerian urban stray cats. PloS one, 7(8), e43621. https://doi.org/10.1371/journal.pone.0043621
Bai, Y., Rizzo, M. F., Alvarez, D., Moran, D., Peruski, L. F., & Kosoy, M. (2015). Coexistence of *Bartonella henselae* and *B. clarridgeiae* in populations of cats and their fleas in Guatemala. Journal of vector ecology: journal of the Society for Vector Ecology, 40(2), 327–332. https://doi.org/10.1111/jvec.12171
Balakrishnan, N., Musulin, S., Varanat, M., Bradley, J. M., & Breitschwerdt, E. B. (2014). Serological and molecular prevalence of selected canine vector borne pathogens in blood donor candidates, clinically healthy volunteers, and stray dogs in North Carolina. Parasites & vectors, 7, 116. https://doi.org/10.1186/1756-3305-7-116
Bergmans, A. M., de Jong, C. M., van Amerongen, G., Schot, C. S., & Schouls, L. M. (1997). Prevalence of *Bartonella* species in domestic cats in The Netherlands. Journal of clinical microbiology, 35(9), 2256–2261. https://doi.org/10.1128/jcm.35.9.2256-2261.1997
Chang, C. C., Lee, C. C., Maruyama, S., Lin, J. W., & Pan, M. J. (2006). Cat-scratch disease in veterinary-associated populations and in its cat reservoir in Taiwan. Veterinary research, 37(4), 565–577. https://doi.org/10.1051/vetres:2006019
Chomel, B. B., Carlos, E. T., Kasten, R. W., Yamamoto, K., Chang, C. C., Carlos, R. S., Abenes, M. V., & Pajares, C. M. (1999). *Bartonella henselae* and *Bartonella clarridgeiae* infection in domestic cats from The Philippines. The American journal of tropical medicine and hygiene, 60(4), 593–597. https://doi.org/10.4269/ajtmh.1999.60.593
Chomel, B. B., Boulouis, H. J., Petersen, H., Kasten, R. W., Yamamoto, K., Chang, C. C., Gandoin, C., Bouillin, C., & Hew, C. M. (2002). Prevalence of *Bartonella* infection in domestic cats in Denmark. Veterinary research, 33(2), 205–213. https://doi.org/10.1051/vetres:2002008
Ebani, V. V., Bertelloni, F., & Fratini, F. (2012). Occurrence of *Bartonella* henselae types I and II in Central Italian domestic cats. Research in veterinary science, 93(1), 63–66. https://doi.org/10.1016/j.rvsc.2011.07.013
Ebani, V. V., Guardone, L., Marra, F., Altomonte, I., Nardoni, S., & Mancianti, F. (2020). Arthropod-Borne Pathogens in Stray Cats from Northern Italy: A Serological and Molecular Survey. Animals: an open access journal from MDPI, 10(12), 2334. https://doi.org/10.3390/ani10122334

Fabbi, M., De Giuli, L., Tranquillo, M., Bragoni, R., Casiraghi, M., & Genchi, C. (2004). Prevalence of *Bartonella henselae* in Italian stray cats: evaluation of serology to assess the risk of transmission of Bartonella to humans. Journal of clinical microbiology, 42(1), 264–268. https://doi.org/10.1128/JCM.42.1.264-268.2004

Fleischman, D. A., Chomel, B. B., Kasten, R. W., Stuckey, M. J., Scarlet, J., Liu, H., Boulouis, H. J., Haddad, N., & Pedersen, N. C. (2015). *Bartonella* Infection among Cats Adopted from a San Francisco Shelter, Revisited. Applied and environmental microbiology, 81(18), 6446–6450. https://doi.org/10.1128/AEM.01864-15

Guptill, L., Wu, C. C., HogenEsch, H., Slater, L. N., Glickman, N., Dunham, A., Syme, H., & Glickman, L. (2004). Prevalence, risk factors, and genetic diversity of *Bartonella henselae* infections in pet cats in four regions of the United States. Journal of clinical microbiology, 42(2), 652–659. https://doi.org/10.1128/JCM.42.2.652-659.2004

Hassan, U. L., Dhaliwal, G. K., Watanabe, M., Ong, B. L., Yakubu, Y., & Tay, S. T. (2017). Feline bartonellosis associated with some clinicopathological conditions in a veterinary hospital in Selangor, Malaysia. Tropical bio medicine, 34(1), 174–179.

Heller, R., Artois, M., Xemar, V., De Briel, D., Gehin, H., Jaulhac, B., Monteil, H., & Piemont, Y. (1997). Prevalence of *Bartonella henselae* and *Bartonella clarridgeiae* in stray cats. Journal of clinical microbiology, 35(6), 1327–1331. https://doi.org/10.1128/jcm.35.6.1327-1331.1997

Inoue, K., Maruyama, S., Kabeya, H., Kawanami, K., Yanai, K., Jitchum, S., & Jittaparapong, S. (2009). Prevalence of *Bartonella* infection in cats and dogs in a metropolitan area, Thailand. Epidemiology and infection, 137(11), 1568–1573. https://doi.org/10.1017/S095026880900257X

Mascarelli, P. E., Tartara, G. P., Pereyra, N. B., & Maggi, R. G. (2016). Detection of *Mycoplasma haemocanis*, *Mycoplasma haematoparvum*, *Mycoplasma suis* and other vector-borne pathogens in dogs from Córdoba and Santa Fé, Argentina. Parasites & vectors, 9(1), 642. https://doi.org/10.1186/s13071-016-1920-8

Melter, O., Hercík, K., Weyant, R. S., Janecek, J., Nemec, A., Mecera, J., Gonzorová, L., & Branny, P. (2003). Detection and characterization of feline Bartonella henselae in the Czech Republic. Veterinary microbiology, 93(3), 261–273. https://doi.org/10.1016/s0378-1135(03)00032-4

Müller, A., Walker, R., Bittencourt, P., Machado, R. Z., Benevenute, J. L., DO Amaral, R. B., Gonçalves, L. R., & André, M. R. (2017). Prevalence, hematological findings and genetic diversity of *Bartonella* spp. in domestic cats from Valdivia, Southern Chile. Parasitology, 144(6), 773–782. https://doi.org/10.1017/S003118201600247X

Pretorius, A. M., Kelly, P. J., Birtles, R. J., & Raoult, D. (1999). Isolation of *Bartonella henselae* from a serologically negative cat in Bloemfontein, South Africa. Journal of the South African Veterinary Association, 70(4), 154–155. https://doi.org/10.4102/jsava.v70i4.785

Rolain, J. M., Locatelli, C., Chabanne, L., Davoust, B., & Raoult, D. (2004). Prevalence of *Bartonella clarridgeiae* and *Bartonella henselae* in domestic cats from France and detection of the organisms in erythrocytes by immunofluorescence. Clinical and diagnostic laboratory immunology, 11(2), 423–425. https://doi.org/10.1128/cdli.11.2.423-425.2003

Sacristán, I., Sieg, M., Acuña, F., Aguilar, E., García, S., López, M. J., Cevidanes, A., Hidalgo-Hermoso, E., Cabello, J., Vahlenkamp, T. W., Millán, J., Poulin, E., & Napolitano, C. (2019). Molecular and serological survey of carnivore pathogens in free-roaming domestic cats of rural communities in southern Chile. The Journal of veterinary medical science, 81(12), 1740–1748. https://doi.org/10.1292/jvms.19-0208

Tobar, B. Z., Lapsley, W. D., Swain, W. L., Jaffe, D. A., Setien, A. A., Galvez-Romero, G., Obregon-Morales, C., Olave-Leyva, J. I., & Chomel, B. B. (2020). *Bartonella* in dogs and fleas from Tulancingo, Hidalgo, Mexico. Medical and veterinary entomology, 34(3), 302–308. https://doi.org/10.1111/mve.12438

Tsai, Y. L., Lin, C. C., Chomel, B. B., Chuang, S. T., Tsai, K. H., Wu, W. J., Huang, C. G., Yu, J. C., Sung, M. H., Kass, P. H., & Chang, C. C. (2011). *Bartonella* infection in shelter cats and dogs and their ectoparasites. Vector borne and zoonotic diseases (Larchmont, N.Y.), 11(8), 1023–1030. https://doi.org/10.1089/vbz.2010.0085

Zhang, X. L., Li, X. W., Li, W. F., Huang, S. J., & Shao, J. W. (2019). Molecular detection and characterization of *Bartonella* spp. in pet cats and dogs in Shenzhen, China. Acta tropica, 197, 105056. https://doi.org/10.1016/j.actatropica.2019.105056

Supplementary -Table 8: Analysis of *Bartonella* spp. infection in cats for subgroup moderators.

Moderator	Subgroup	studies (number)	Prevalence (%) (95% CI)	P-value	OR	Lower	upper	Q-value	df	I^2	T au ^{2}	T au
Lifestyle	Free roaming	18	21.2 (19.5- 22.9)	< **0.0001**	5.7	2.9	9.01	102.18	17	86.36	1.1	1.03
	Indoor		6.7 (5.7- 7.5)									
Age	≤ 12m	17	26.9 (24.4- 29.5)	**0.001**	1.64	1.2	2.2	29.03	16	44.89	0.14	0.4
	> 12m		15.8 (14.7- 16.9)									
Gender	M	26	11.1% (10.2- 12.1%)	0.63		0.7	1.01	22.9	25	0.00		
	F		12.1% (11.2- 13%)									
Ectoparasite infestation	Yes	12	28.5 (25.7- 31.4)	**0.004**	1.95	1.00	3.7	29.3	11	62.4	0.5	0.7
	No		14.6 (12.7- 16.7)									

Supplementary-Figure 1: Forest plot of the random-effects meta-analysis of *Bartonella* prevalence estimates in cat populations for individual studies. Inverse variance index (I^2= 95.8%), variance between studies ($\tau 2$ =0.913), p value < 0.0001, Q-value = 2064.9.

Bartonella spp. prevalence in cats

Authors	Country	Positive cases	Sample size	Statistics for each study: Event rate	Lower limit	Upper limit	Z-Value	p-Value
Gracia et al., 2015	Spain	0	89	0.006	0.000	0.083	-3.658	0.000
Bessas et al., 2016	Algeria	1	107	0.009	0.001	0.063	-4.642	0.000
Barradas et al., 2019	Angola	1	100	0.010	0.001	0.068	-4.572	0.000
Tabar et al., 2008	Spain	1	100	0.010	0.001	0.068	-4.572	0.000
Braga et al., 2015	Brazil	3	182	0.016	0.005	0.050	-7.023	0.000
Miceli et al.,2013	Brazil	4	178	0.022	0.008	0.058	-7.460	0.000
Latrofa et al.,2020	Italy	24	958	0.025	0.017	0.037	-17.711	0.000
Saengsawang et al.,2021	Thailand	13	513	0.025	0.015	0.043	-12.991	0.000
Dybing et al ., 2016	Australia	3	116	0.026	0.008	0.077	-6.203	0.000
Maia et al., 2014	Portugal	19	649	0.029	0.019	0.045	-15.037	0.000
Mesquita et al.,. 2021	Angola	2	66	0.030	0.008	0.113	-4.826	0.000
Pretorius et al., 1999	South Africa	1	31	0.032	0.005	0.196	-3.346	0.001
Otranto et al., 2017	Italy	13	330	0.039	0.023	0.067	-11.287	0.000
Zhang et al.,2019	China	8	203	0.039	0.020	0.077	-8.853	0.000
Bortoli et al., 2012	Brazil	2	46	0.043	0.011	0.158	-4.275	0.000
Braga et al., 2012	Brazil	9	200	0.045	0.024	0.084	-8.957	0.000
Sato et al., 2017	Japan	80	1754	0.046	0.037	0.056	-26.572	0.000
Razgunaite et al., 2021	Lithuania	8	163	0.049	0.025	0.095	-8.175	0.000
Juvet et al., 2010	Ireland	6	116	0.052	0.023	0.110	-6.938	0.000
Zobba et al.,2009	Italy	3	55	0.055	0.018	0.156	-4.804	0.000
Satranarakun et al .,2016	Thailand	11	200	0.055	0.031	0.097	-9.169	0.000
Chaloner et al., 2011	UK	103	1782	0.058	0.048	0.070	-27.497	0.000
Millán et al., 2016	Spain	2	34	0.059	0.015	0.207	-3.804	0.000
Maruyama et al., 2000	Japan	50	690	0.072	0.055	0.094	-17.362	0.000
Kamrani et al., 2008	Canada	51	691	0.074	0.057	0.096	-17.386	0.000
Lobetti & Lappin 2012	South Africa	8	102	0.078	0.040	0.149	-6.690	0.000
Rolain et al., 2004	France	8	99	0.081	0.041	0.153	-6.593	0.000
Melter et al., 2003	Czech Republic	5	61	0.082	0.035	0.182	-5.176	0.000
Mylonakis et al.,2018	Greece	8	94	0.085	0.043	0.161	-6.425	0.000
Zhang et al., 2021	China	57	668	0.085	0.066	0.109	-17.127	0.000
Alanazi et al., 2020	Saudi Arabia	4	44	0.091	0.035	0.218	-4.391	0.000
Srisanyong et al., 2016	Thailand	13	139	0.094	0.055	0.154	-7.797	0.000
Sacristán et al .,2019	Chile	3	30	0.100	0.033	0.268	-3.610	0.000
Shannon et al., 2017	USA	5	49	0.102	0.043	0.223	-4.608	0.000
Ebani et al., 2012	Italy	24	234	0.103	0.070	0.148	-10.066	0.000
Arvand et al., 2001	Germany	20	193	0.104	0.068	0.155	-9.135	0.000
Attipa et al., 2017	Cyprus	19	174	0.109	0.071	0.165	-8.635	0.000
Yuan et al., 2011	China	46	359	0.128	0.097	0.167	-12.144	0.000
Pedrassani et al., 2019	Brazil	4	30	0.133	0.051	0.306	-3.485	0.000
Angioni et al., 2020	Italy	7	52	0.135	0.066	0.256	-4.580	0.000
Inoue et al., 2009	Thailand	47	312	0.151	0.115	0.195	-10.928	0.000
Huang et al., 2019	China	25	164	0.152	0.105	0.216	-7.897	0.000
Mietze et al., 2011	Germany	28	169	0.166	0.117	0.230	-7.813	0.000
Joseph et al., 1997	New Zealand	8	48	0.167	0.086	0.299	-4.156	0.000
Hassan et al., 2017	Malysia	48	284	0.169	0.130	0.217	-10.058	0.000
Assarasakorn et al., 2012	Thailand	26	153	0.170	0.118	0.238	-7.368	0.000
Solano-Gallego et al., 2006	Spain	8	47	0.170	0.087	0.305	-4.081	0.000
Staggemeier et al., 2010	Brazil	8	47	0.170	0.087	0.305	-4.081	0.000
Azzag et al., 2012	Algeria	36	211	0.171	0.126	0.227	-8.640	0.000
Cicuttin et al., 2014	Argentina	18	101	0.178	0.115	0.265	-5.879	0.000
Müller et al., 2017	Chile	67	370	0.181	0.145	0.224	-11.178	0.000
Fabbi et al., 2004	Italy	140	769	0.182	0.156	0.211	-16.078	0.000
Chang et al., 2006	Taiwan	25	131	0.191	0.132	0.267	-6.497	0.000
Malheiros et al., 2016	Brazil	6	30	0.200	0.093	0.379	-3.037	0.002
Tsai et al., 2011	Taiwan	21	103	0.204	0.137	0.293	-5.570	0.000
Persichetti et al., 2018	Italy	42	197	0.213	0.162	0.276	-7.506	0.000
Bergmans et al., 1997	Netherlands	25	113	0.221	0.154	0.307	-5.553	0.000
Ravicini et al.,2016	Spain	26	116	0.224	0.157	0.309	-5.577	0.000
Chomel et al., 2002	Denmark	21	93	0.226	0.152	0.322	-4.968	0.000
Guptill et al., 2004	USA	65	271	0.240	0.193	0.294	-8.108	0.000
Silva et al., 2019	Brazil	22	89	0.247	0.169	0.347	-4.532	0.000
Gutiérrez et al.,2013	Israel	84	334	0.251	0.208	0.301	-8.648	0.000
Staggemeier et al., 2014	Brazil	12	47	0.255	0.151	0.398	-3.200	0.001
Ebani et al., 2020	Italy	23	85	0.271	0.187	0.374	-4.062	0.000
Fleischman et al., 2015	USA	95	351	0.271	0.227	0.320	-8.252	0.000
Maruyama et al., 2001	Thailand	76	275	0.276	0.227	0.332	-7.138	0.000
André et al., 2014	Brazil	11	37	0.297	0.173	0.461	-2.392	0.017
Chekli et al., 2021	Morocco	39	130	0.300	0.228	0.384	-4.427	0.000
André et al., 2016	Brazil	46	151	0.305	0.237	0.383	-4.668	0.000
Bai et al., 2015	Guatemala	48	142	0.338	0.265	0.420	-3.789	0.000
Grippi et al., 2021	Italy	148	429	0.345	0.301	0.391	-6.313	0.000
Hwang et al., 2018	Korea	105	302	0.348	0.296	0.403	-5.208	0.000
Branley et al., 1996	Australia	27	77	0.351	0.253	0.463	-2.580	0.010
Persichetti et al., 2016	Italy	16	42	0.381	0.248	0.534	-1.528	0.127
Chomel et al.,1997	USA	81	205	0.395	0.331	0.464	-2.981	0.003
Huang et al., 2019	Caribbean	58	146	0.397	0.321	0.479	-2.465	0.014
Furquim et al.,2021	Brazil	122	306	0.399	0.345	0.455	-3.519	0.000
Raimundo et al., 2019	Brazil	83	208	0.399	0.335	0.467	-2.892	0.004
Karakavuk et al., 2021	Turkey	67	167	0.401	0.330	0.477	-2.537	0.011
Kim et al., 2009	Korea	59	146	0.404	0.328	0.486	-2.303	0.021
Mazurek et al., 2020	Poland	272	672	0.405	0.368	0.442	-4.907	0.000
Crissiuma et al., 2011	Brazil	17	40	0.425	0.283	0.580	-0.945	0.345
Hwang & Gottdenker et al., 2013	USA	16	37	0.432	0.284	0.594	-0.819	0.413
Heller et al ., 1997	France	50	94	0.532	0.431	0.630	0.618	0.536
Lappin et al., 2006	USA	51	92	0.554	0.452	0.652	1.041	0.298
Levy et al., 2008	Ecuador	31	52	0.596	0.459	0.720	1.378	0.168
Chomel et al.,1999	Philippines	19	31	0.613	0.435	0.765	1.246	0.213
Gutiérrez et al.,2015	Israel	23	36	0.639	0.473	0.777	1.644	0.100
				0.153	0.127	0.182	-15.764	0.000

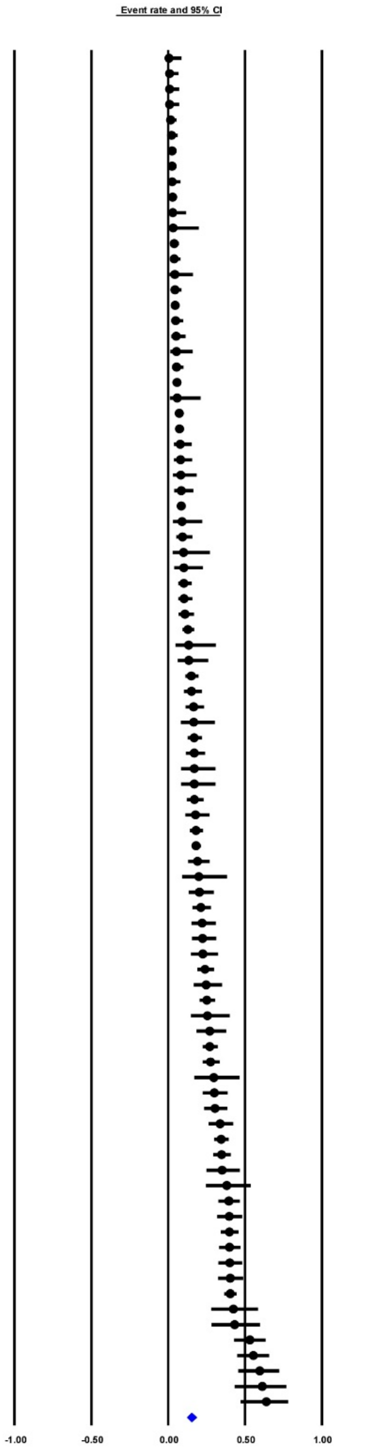

Supplementary-Figure 2: Forest plot of the random-effects meta-analysis of *Bartonella* prevalence estimates in dog populations for individual studies. Inverse variance index (I^2=87.7%), variance between studies ($\tau2$ =1.186), *P*-value < 0.0001, Q-value = 464.8.

Bartonella spp. prevalence in dogs

Authors	Country	Positive cases	Sample size	Statistics for each study: Event rate	Lower limit	Upper limit	Z-Value	p-Value
Maia et al., 2015	Portugal	0	1010	0.000	0.000	0.008	-5.381	0.000
Suh et al., 2017	Korea	0	532	0.001	0.000	0.015	-4.927	0.000
Pérez Vera et al ., 2014	Finland	0	350	0.001	0.000	0.022	-4.630	0.000
Saengsawang et al.,2021	Thailand	0	295	0.002	0.000	0.026	-4.509	0.000
Zhang et al., 2019	China	0	272	0.002	0.000	0.029	-4.451	0.000
Otranto et al., 2017	Italy	0	263	0.002	0.000	0.030	-4.427	0.000
Diniz et al., 2010	USA	0	233	0.002	0.000	0.033	-4.341	0.000
Abdullah et al., 2021	Egypt	0	206	0.002	0.000	0.037	-4.254	0.000
Zobba et al., 2009	italy	0	190	0.003	0.000	0.040	-4.197	0.000
Inoue et al., 2009	Thailand	1	350	0.003	0.000	0.020	-5.847	0.000
Chekli et al., 2020	Morocco	0	153	0.003	0.000	0.050	-4.043	0.000
Lauzi et al., 2016	Maio Island	0	153	0.003	0.000	0.050	-4.043	0.000
Tabar et al., 2009	Spain	0	153	0.003	0.000	0.050	-4.043	0.000
Cevidanes et al., 2018	Chile	0	153	0.003	0.000	0.050	-4.043	0.000
Silva et al., 2019	Brazil	0	124	0.004	0.000	0.061	-3.894	0.000
Rymaszewska et al., 2011	Poland	1	242	0.004	0.001	0.029	-5.473	0.000
Huggins et al., 2021	Cambodia	2	467	0.004	0.001	0.017	-7.689	0.000
Cicuttin et al., 2014	Argentina	0	94	0.005	0.000	0.079	-3.697	0.000
Welc-Faleciak et al. 2009	Poland	0	82	0.006	0.000	0.089	-3.600	0.000
Alanazi et al., 2020	Saudi Arabia	1	144	0.007	0.001	0.048	-4.946	0.000
Henriques et al., 2021	Portugal	0	61	0.008	0.001	0.116	-3.389	0.001
Bessas et al., 2016	Algeria	1	117	0.009	0.001	0.058	-4.733	0.000
Ereqat et al., 2016	Palestine	1	110	0.009	0.001	0.062	-4.670	0.000
Huggins et al., 2019	Thailand	1	100	0.010	0.001	0.068	-4.572	0.000
Diniz et al., 2007a	Brazil	2	198	0.010	0.003	0.039	-6.451	0.000
Kidd et al., 2017	USA	0	42	0.012	0.001	0.160	-3.123	0.002
Proboste et al., 2015	Uganda	0	38	0.013	0.001	0.175	-3.052	0.002
Millán et al., 2016	Spain	0	37	0.013	0.001	0.178	-3.033	0.002
Tsai et al., 2011	Taiwan	2	120	0.017	0.004	0.064	-5.718	0.000
Billeter etal., 2012	Thailand	3	164	0.018	0.006	0.055	-6.835	0.000
Henn et al., 2007	USA	4	182	0.022	0.008	0.057	-7.507	0.000
Mesquita et al., 2021	Angola	0	19	0.025	0.002	0.298	-2.558	0.011
Clarke et al., 2014	Ghana	0	17	0.028	0.002	0.322	-2.479	0.013
Mascarelli et al., 2016	Argentina	2	66	0.030	0.008	0.113	-4.826	0.000
Singer et al., 2020	Philippines	6	160	0.038	0.017	0.081	-7.799	0.000
Diniz et al., 2009	Greece	2	50	0.040	0.010	0.146	-4.404	0.000
Müller et al., 2018	Chile	6	139	0.043	0.020	0.093	-7.424	0.000
Otranto et al., 2010	Italy	6	109	0.055	0.025	0.117	-6.769	0.000
Kernif et al., 2010	Algeria	5	80	0.063	0.026	0.142	-5.863	0.000
Azzag et al., 2015	Algeria	7	96	0.073	0.035	0.145	-6.478	0.000
Benner et al., 2013	Colombia	2	22	0.091	0.023	0.300	-3.105	0.002
Celebi et al., 2010	Turkey	23	250	0.092	0.062	0.135	-10.463	0.000
Pérez et al., 2011	USA	61	663	0.092	0.072	0.116	-17.038	0.000
Tobar et al., 2020	Mexico	3	31	0.097	0.032	0.261	-3.677	0.000
Diniz et al., 2013	Peru	21	205	0.102	0.068	0.152	-9.423	0.000
Diniz et al., 2009	Italy	7	60	0.117	0.057	0.225	-5.034	0.000
Samsami et al., 2020	Iran	12	98	0.122	0.071	0.203	-6.391	0.000
Levy et al., 2008	Ecuador	13	95	0.137	0.081	0.222	-6.170	0.000
Belkhiria et al., 2017	Tunisia	22	149	0.148	0.099	0.214	-7.592	0.000
Arenas et al., 2019	Mexico	3	19	0.158	0.052	0.392	-2.661	0.008
Far et al., 2021	Jordan	14	80	0.175	0.106	0.274	-5.270	0.000
Balakrishnan et al., 2014	USA	21	118	0.178	0.119	0.258	-6.358	0.000
Varanat et al., 2011	USA	28	137	0.204	0.145	0.280	-6.415	0.000
Ebani et al., 2015	Italy	24	117	0.205	0.141	0.288	-5.916	0.000
Kim et al., 2009	Korea	13	54	0.241	0.145	0.372	-3.609	0.000
Greco et al., 2019	Iran	16	66	0.242	0.154	0.360	-3.967	0.000
Bai et al., 2010	Thailand	60	192	0.313	0.251	0.381	-5.064	0.000
Chomel et al., 2012	Iraq	36	97	0.371	0.281	0.471	-2.509	0.012
				0.036	0.026	0.051	-18.353	0.000

Event rate and 95% CI

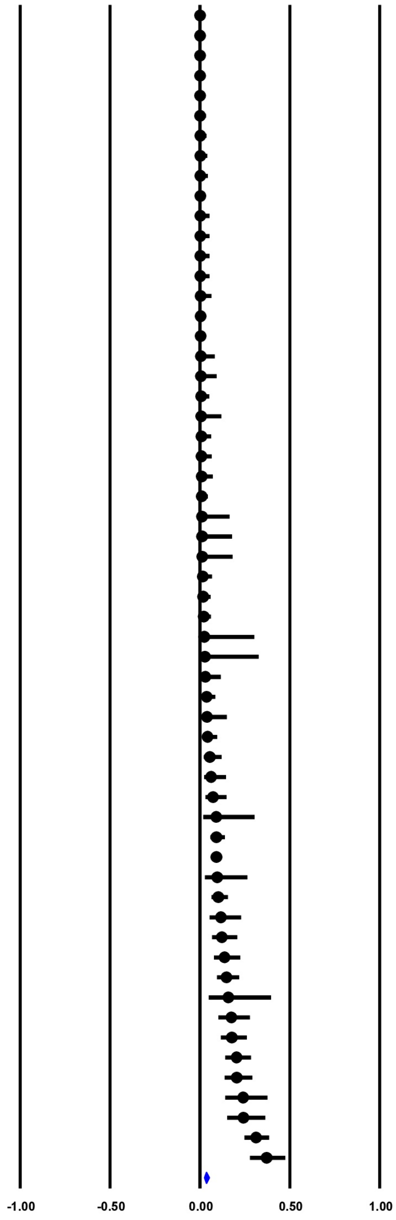

Supplementary-Figure 3. Meta-analysis of *Bartonella* species in cats based on category of countrywide location coordinates

Prevalence of Bartonella species in cats according to countrywide location coordinates

Group by Subgroup within study	Study name	Subgroup within study	Event rate	Lower limit	Upper limit	Z-Value	p-Value
first band	Angola	first band	0.018	0.006	0.055	-6.857	0.000
first band	Spain	first band	0.096	0.070	0.130	-12.980	0.000
first band	Algeria	first band	0.116	0.085	0.156	-11.593	0.000
first band	Germany	first band	0.133	0.101	0.172	-12.119	0.000
first band	Italy	first band	0.140	0.128	0.152	-35.378	0.000
first band	Portugal	first band	0.029	0.019	0.045	-15.037	0.000
first band	Netherlands	first band	0.221	0.154	0.307	-5.553	0.000
first band	Denmark	first band	0.226	0.152	0.322	-4.968	0.000
first band	Morocco	first band	0.300	0.228	0.384	-4.427	0.000
first band	France	first band	0.301	0.240	0.369	-5.381	0.000
first band	Guatemala	first band	0.338	0.265	0.420	-3.789	0.000
first band	Poland	first band	0.405	0.368	0.442	-4.907	0.000
first band	Philippines	first band	0.613	0.435	0.765	1.246	0.213
first band	Ireland	first band	0.052	0.023	0.110	-6.938	0.000
first band	UK	first band	0.058	0.048	0.070	-27.497	0.000
first band	Czech Republic	first band	0.082	0.035	0.182	-5.176	0.000
first band			0.151	0.100	0.220	-7.264	0.000
fourth band	Saudi Arabia	fourth band	0.091	0.035	0.218	-4.391	0.000
fourth band	China	fourth band	0.098	0.083	0.114	-24.645	0.000
fourth band	Thailand	fourth band	0.117	0.102	0.134	-25.925	0.000
fourth band	Australia	fourth band	0.155	0.111	0.214	-8.520	0.000
fourth band	New Zealand	fourth band	0.167	0.086	0.299	-4.156	0.000
fourth band	Malaysia	fourth band	0.169	0.130	0.217	-10.058	0.000
fourth band	Chile	fourth band	0.175	0.141	0.215	-11.784	0.000
fourth band	Argentina	fourth band	0.178	0.115	0.265	-5.879	0.000
fourth band	Taiwan	fourth band	0.197	0.151	0.252	-8.558	0.000
fourth band	Brazil	fourth band	0.219	0.200	0.240	-20.953	0.000
fourth band	USA	fourth band	0.311	0.284	0.341	-11.647	0.000
fourth band	Korea	fourth band	0.366	0.323	0.412	-5.599	0.000
fourth band	Saint Kitts	fourth band	0.397	0.321	0.479	-2.465	0.014
fourth band	Ecuador	fourth band	0.596	0.459	0.720	1.378	0.168
fourth band	Japan	fourth band	0.053	0.045	0.063	-31.943	0.000
fourth band	Canada	fourth band	0.074	0.057	0.096	-17.386	0.000
fourth band			0.183	0.131	0.250	-7.416	0.000
second band	Greece	second band	0.085	0.043	0.161	-6.425	0.000
second band	Lithuania	second band	0.049	0.025	0.095	-8.175	0.000
second band	South Africa	second band	0.068	0.036	0.125	-7.598	0.000
second band			0.066	0.045	0.095	-12.827	0.000
third band	Cyprus	third band	0.109	0.071	0.165	-8.635	0.000
third band	Israel	third band	0.289	0.245	0.337	-7.843	0.000
third band	Turkey	third band	0.401	0.330	0.477	-2.537	0.011
third band			0.248	0.133	0.416	-2.828	0.005
Overall			0.131	0.107	0.159	-16.072	0.000

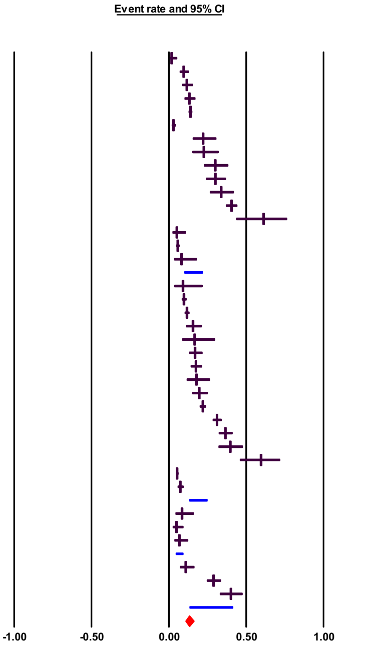

Supplementary-Figure 4: Meta-analysis of *Bartonella* species in dogs based on category of countrywide location coordinates.

Prevalence of Bartonella species in dogs according to countrywide location coordinates

Group by Subgroup within study		Subgroup within study	Event rate	Lower limit	Upper limit	Z-Value	p-Value
first band	Portugal	first band	0.000	0.000	0.007	-5.422	0.000
first band	Angola	first band	0.025	0.002	0.298	-2.558	0.011
first band	Ghana	first band	0.028	0.002	0.322	-2.479	0.013
first band	Algeria	first band	0.044	0.026	0.075	-10.820	0.000
first band	Italy	first band	0.050	0.036	0.068	-17.448	0.000
first band	Tunisia	first band	0.148	0.099	0.214	-7.592	0.000
first band	Spain	first band	0.003	0.000	0.040	-4.197	0.000
first band	Poland	first band	0.003	0.000	0.022	-5.769	0.000
first band	Morocco	first band	0.003	0.000	0.050	-4.043	0.000
first band			0.022	0.009	0.051	-8.529	0.000
fourth band	Brazil	fourth band	0.006	0.002	0.024	-7.155	0.000
fourth band	Saudi Arabia	fourth band	0.007	0.001	0.048	-4.946	0.000
fourth band	Argentina	fourth band	0.013	0.003	0.049	-6.141	0.000
fourth band	Taiwan	fourth band	0.017	0.004	0.064	-5.718	0.000
fourth band	Chile	fourth band	0.021	0.009	0.045	-9.368	0.000
fourth band	Korea	fourth band	0.022	0.013	0.038	-13.498	0.000
fourth band	Philippine	fourth band	0.038	0.017	0.081	-7.799	0.000
fourth band	srilanka	fourth band	0.051	0.016	0.146	-4.939	0.000
fourth band	Thailand	fourth band	0.059	0.047	0.075	-21.653	0.000
fourth band	USA	fourth band	0.083	0.069	0.099	-24.575	0.000
fourth band	Colombia	fourth band	0.091	0.023	0.300	-3.105	0.002
fourth band	Peru	fourth band	0.102	0.068	0.152	-9.423	0.000
fourth band	China	fourth band	0.002	0.000	0.029	-4.451	0.000
fourth band	Mexico	fourth band	0.120	0.055	0.242	-4.578	0.000
fourth band	Ecuador	fourth band	0.137	0.081	0.222	-6.170	0.000
fourth band	Iran	fourth band	0.171	0.121	0.236	-7.616	0.000
fourth band	Iraq	fourth band	0.371	0.281	0.471	-2.509	0.012
fourth band	Cambodia	fourth band	0.004	0.001	0.017	-7.689	0.000
fourth band			0.047	0.029	0.074	-12.113	0.000
second band	Finland	second band	0.001	0.000	0.022	-4.630	0.000
second band	Greece	second band	0.040	0.010	0.146	-4.404	0.000
second band	Maio island	second band	0.003	0.000	0.050	-4.043	0.000
second band			0.007	0.001	0.068	-4.218	0.000
third band	Palestine	third band	0.009	0.001	0.062	-4.670	0.000
third band	Uganda	third band	0.013	0.001	0.175	-3.052	0.002
third band	Turkey	third band	0.092	0.062	0.135	-10.463	0.000
third band	Jordan	third band	0.175	0.106	0.274	-5.270	0.000
third band	Egypt	third band	0.002	0.000	0.037	-4.254	0.000
third band			0.044	0.015	0.123	-5.469	0.000
Overall			0.038	0.026	0.055	-16.206	0.000

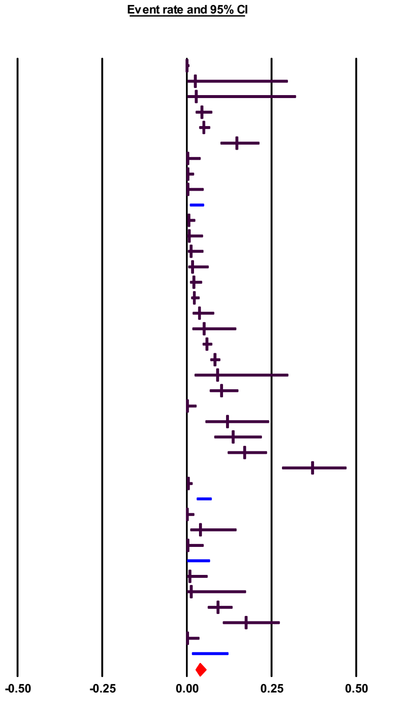

Supplementary-Figure 5: *Meta-analysis of Bartonella spp. from cats based on individual study/sampling site location coordinates. (reported as individual file due to the high size of the figure)*

Supplementary-Figure 6: *Meta-analysis of Bartonella spp. from dogs based on category of individual study/sampling site location coordinates. (reported as individual file due to the high size of the figure)*

Supplementary-Figure 7: Forest plot of the random-effect meta-analysis on the association of *B. henselae* genotypes I and II and continental distributions. Inverse variance index (I^2=92.865), variance between studies ($\tau 2$ =3.037), *P*- value < 0.0001, Q-value = 168.186.

Continental distribution of B. henselae genotypes

Study name	Subgroup within study	Statistics for each study				
		Odds ratio	Lower limit	Upper limit	Z-Value	p-Value
Europe	Combined	2.835	2.020	3.979	6.027	0.000
Asia	Combined	0.031	0.016	0.058	-10.847	0.000
Africa	Combined	0.508	0.181	1.424	-1.288	0.198
America	Combined	0.859	0.585	1.259	-0.781	0.435
		0.447	0.074	2.708	-0.875	0.381

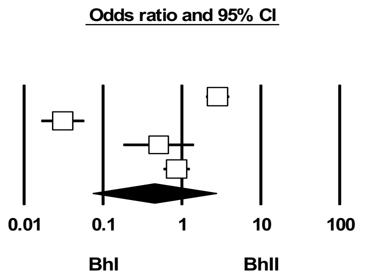

Meta Analysis

Supplementary-Figure 8: Forest plot of the random-effect meta-analysis on the association of *Bartonella* prevalence estimates and different moderators.

Panel a: *Bartonella* spp. prevalence estimates associated with age (P=0.001, I^2=44.89)
Panel b: *Bartonella* spp. prevalence estimates associated with gender (P=0.63)
Panel c: *Bartonella* spp. prevalence estimates associated with ectoparasite infestation (P=0.004, I^2=62.4)
Panel d: *Bartonella* spp. prevalence estimates association with lifestyle (P<0.0001, I^2=86.36)

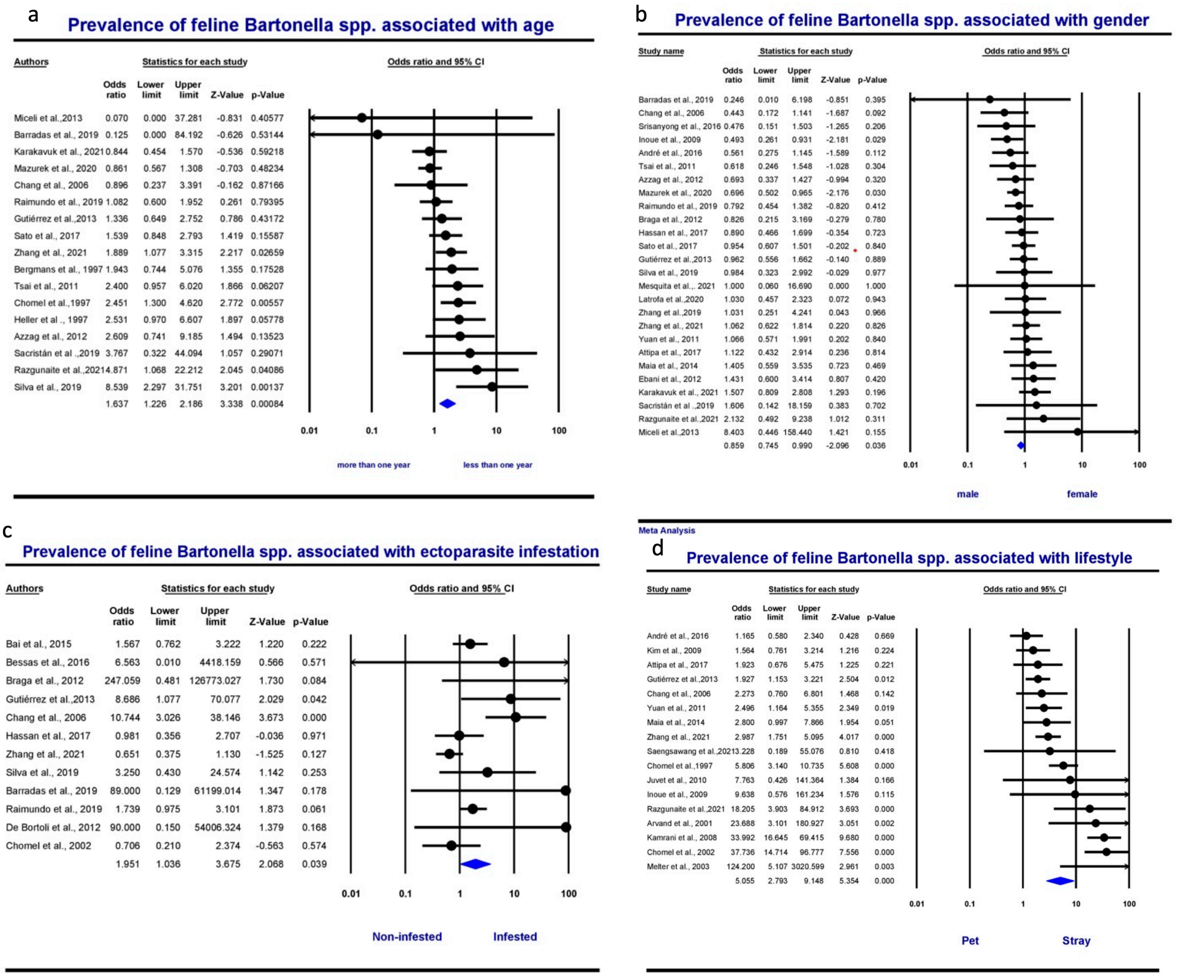

Supplement: Supplementary Materials — Table S1: list of the studies (n.88) included in this study reporting the Bartonella spp. prevalence estimates of cat populations. Table S2: list of the studies (n.58) included in this study reporting the Bartonella spp. prevalence estimates of dogs' populations. Reference list of the studies submitted to meta-analyses as detailed in Tables S1 and S2. Table S3: meta-regression analyses of the prevalence estimates of Bartonella spp. in cat populations according to continent and subregion stratifications. Table S4: meta-regression analyses of the prevalence estimates of Bartonella spp. in dog populations according to continent and subregion stratifications. Table S5: summary of purely spatial scan statistics using the Bernoulli probability model (only significant clusters reported). Table S6: pooled prevalence estimates of Bartonella species according to animal host species and continent. Table S7: continental distribution of B. henselae genotypes detected from cats and dogs. Table 7.1: reference list of the studies reporting the genotype characterization of B. henselae detected in both cats and dogs and submitted to the analyses as reported in Supplementary Table 7. Table S8: analysis of Bartonella spp. infection in cats for subgroup moderators. Figure S1: forest plot of the random-effects meta-analysis of Bartonella prevalence estimates in cat populations for individual studies. Inverse variance index (I2 = 95.8%), variance between studies (τ2 = 0.913), p-value <0.0001, Q-value = 2064.9. Figure S2: forest plot of the random-effects meta-analysis of Bartonella prevalence estimates in dog populations for individual studies. Inverse variance index (I2 = 87.7%), variance between studies (τ2 = 1.186), p-value <0.0001, Q-value = 464.8. Figure S3: meta-analysis of Bartonella spp. prevalence estimates in cats based on the category of countrywide location coordinates. Figure S4: meta-analysis of Bartonella spp. prevalence estimates in dogs based on the category of countrywid [file 7867562.f1.pdf]
